# Pharmacological Characterization of Low Molecular Weight Biased Agonists at the Follicle Stimulating Hormone Receptor

**DOI:** 10.3390/ijms22189850

**Published:** 2021-09-12

**Authors:** Francesco De Pascali, Mohammed Akli Ayoub, Riccardo Benevelli, Silvia Sposini, Jordan Lehoux, Nathalie Gallay, Pauline Raynaud, Flavie Landomiel, Frédéric Jean-Alphonse, Christophe Gauthier, Lucie P. Pellissier, Pascale Crépieux, Anne Poupon, Asuka Inoue, Nicolas Joubert, Marie-Claude Viaud-Massuard, Livio Casarini, Manuela Simoni, Aylin C. Hanyaloglu, Selva G. Nataraja, Henry N. Yu, Stephen S. Palmer, Romain Yvinec, Eric Reiter

**Affiliations:** 1 Physiologie de la Reproduction et des Comportements (PRC), Institut National de Recherche pour l’Agriculture, l’Alimentation et l’Environnement (INRAE), Centre National de la Recherche Scientifique (CNRS), Institut Français du Cheval et de l’Equitation (IFCE), Université de Tours, 37380 Nouzilly, France; francesco.depascali@jefferson.edu (F.D.P.); mayoub@uaeu.ac.ae (M.A.A.); N.LANGONNE@chu-tours.fr (N.G.); pauline.raynaud@inrae.fr (P.R.); flavie.landomiel@gmail.com (F.L.); Frederic.Jean-Alphonse@inrae.fr (F.J.-A.); christophe.gauthier@inrae.fr (C.G.); lucie.pellissier@inrae.fr (L.P.P.); pascale.crepieux@inrae.fr (P.C.); Anne.Poupon@inrae.fr (A.P.); 2 Biology Department, College of Science, United Arab Emirates University, Al Ain 15551, United Arab Emirates; 3 Unit of Endocrinology, Department of Biomedical, Metabolic and Neural Sciences, University of Modena and Reggio Emilia, 41125 Modena, Italy; riccardo.benevelli95@gmail.com (R.B.); livio.casarini@unimore.it (L.C.); manuela.simoni@unimore.it (M.S.); 4 Institute of Reproductive and Developmental Biology, Department of Metabolism, Digestion and Reproduction, Imperial College London, London SW7 2AZ, UK; silvia.sposini@u-bordeaux.fr (S.S.); a.hanyaloglu@imperial.ac.uk (A.C.H.); 5 GICC EA7501, Université de Tours, 37032 Tours, France; jordan.lehoux.chem@gmail.com (J.L.); nicolas.joubert@univ-tours.fr (N.J.); marieclaude.viaudmassuard@mcsaf.fr (M.-C.V.-M.); 6 Inria Saclay-Île-de-France, Inria, Université Paris-Saclay, 91120 Palaiseau, France; 7 Graduate School of Pharmaceutical Sciences, Tohoku University, Sendai 980-8578, Japan; iaska@tohoku.ac.jp; 8 TocopheRx Inc., Burlington, MA 01803, USA; selvaboston@yahoo.com (S.G.N.); henry@canwellpharma.com (H.N.Y.); Stephen.Palmer2@bcm.edu (S.S.P.)

**Keywords:** FSHR, biased signaling, allosteric ligands, system bias, operational model

## Abstract

Follicle-stimulating hormone receptor (FSHR) plays a key role in reproduction through the activation of multiple signaling pathways. Low molecular weight (LMW) ligands composed of biased agonist properties are highly valuable tools to decipher complex signaling mechanisms as they allow selective activation of discrete signaling cascades. However, available LMW FSHR ligands have not been fully characterized yet. In this context, we explored the pharmacological diversity of three benzamide and two thiazolidinone derivatives compared to FSH. Concentration/activity curves were generated for Gαs, Gαq, Gαi, β-arrestin 2 recruitment, and cAMP production, using BRET assays in living cells. ERK phosphorylation was analyzed by Western blotting, and CRE-dependent transcription was assessed using a luciferase reporter assay. All assays were done in either wild-type, Gαs or β-arrestin 1/2 CRISPR knockout HEK293 cells. Bias factors were calculated for each pair of read-outs by using the operational model. Our results show that each ligand presented a discrete pharmacological efficacy compared to FSH, ranging from super-agonist for β-arrestin 2 recruitment to pure Gαs bias. Interestingly, LMW ligands generated kinetic profiles distinct from FSH (i.e., faster, slower or transient, depending on the ligand) and correlated with CRE-dependent transcription. In addition, clear system biases were observed in cells depleted of either Gαs or β-arrestin genes. Such LMW properties are useful pharmacological tools to better dissect the multiple signaling pathways activated by FSHR and assess their relative contributions at the cellular and physio-pathological levels.

## 1. Introduction

Follicle-stimulating hormone receptor (FSHR), together with its cognate endogenous ligand follicle-stimulating hormone (FSH), plays a key role in regulating gametogenesis in both female and male reproduction [1,2]. FSHR is a G protein-coupled receptor (GPCR) made of a large glycosylated extracellular domain (ECD) containing multiple leucine-rich repeats connected to seven spanning transmembrane (TMD) α-helices joined together by three extracellular loops and three intracellular loops and terminated by a C-terminal intracellular tail. The horse-shoe-shaped ECD is prototypical to the glycoprotein hormone receptor subfamily where the orthosteric binding site is located [3]. Once activated, FSHR induces a plethora of intracellular mechanisms [4,5], amongst which are the Gα_s_/PKA/cAMP [6,7] and extracellular signal-regulated kinase (ERK) pathways [8]. Like most GPCRs, β-arrestin recruitment and binding to FSHR lead to desensitization and internalization [9] while also assembling signaling modules [10,11,12]. In addition to Gα_s_, FSHR displays coupling promiscuity [13] with Gα_q/11_ [14] and Gα_i_ [15]. The FSHR/FSH system is implicated in several reproduction-related pathologies both in women and men. These include premature ovarian insufficiency (POI), polycystic ovarian syndrome (PCOS), and male idiopathic infertility. Another severe condition is ovarian hyperstimulation syndrome (OHSS). Indeed, FSHR is the target of assisted reproductive techniques (ART), in which ovulation is induced through the injection of FSH preparations in a procedure called controlled ovarian stimulation. This procedure may lead to OHSS. Despite the fact that all aforementioned pathologies are well known and clinically described, the active role of FSHR, as well as the molecular mechanisms causing these disorders, are not entirely known. FSH induces multiple signaling pathways upon FSHR activation, making it difficult to understand which specific signaling module may be implicated in a specific pathology. In recent years, a growing body of evidence is highlighting the importance of dissecting GPCRs signaling pathways for two main reasons. (1) In many cases, a particular GPCR-associated pathology is driven by a specific signaling pathway, and (2) current drugs are balanced agonists that result in both beneficial and unwanted side effects. We believe that having molecules capable of biasing signaling at the FSHR constitutes an important pharmacological tool to understand the specific FSHR signaling pathway contributing to pathologies (such as POI, PCOS) as well as to develop new generation drugs devoid of the adverse side effects of current clinical treatments (OHSS) [16].

Accumulating structural, biophysical, and pharmacological evidence has transformed our understanding of GPCR activation and pharmacological modulation. The fact that multiple active and inactive receptor conformations co-exist is supported by overwhelming structural and biophysical evidence [17,18,19]. Consequently, efficacy is now considered multi-dimensional and explicitly incorporates the notion that receptors can engage distinct subsets of their full signaling repertoire [20]. This implies that different subsets of conformations can be stabilized by different ligands with distinct transduction mechanisms. This is the concept of pharmacological bias [21,22,23]. This class of ligands can modulate signaling in the presence (positive/negative allosteric modulators) or in the absence (allosteric agonists) of the endogenous ligand. Importantly, synthetic allosteric modulators of GPCRs can also lead to pharmacological bias [24,25]. It is also established that ligand bias must be set apart from system bias or observational bias [26].

**Figure 1:**
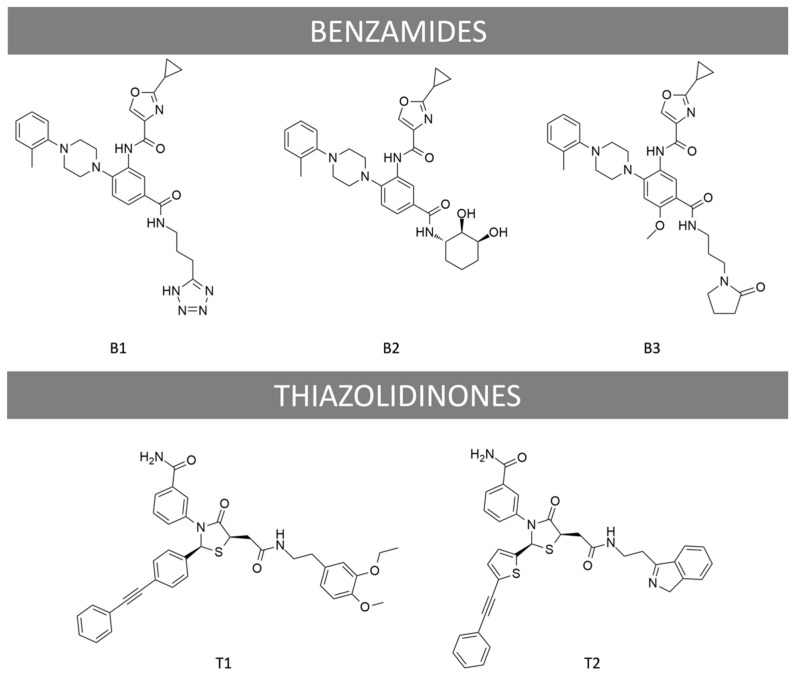
Chemical structures of LMW ligands used in the study. B1, B2, B3 are Benzamide derivatives; T1 is a Thiazolidinone derivative, and T2 is an antagonist obtained by chemically modifying T1.

To date, different classes of low molecular weight (LMW) molecules capable of modulating FSHR have been identified [27,28], including negative allosteric modulators (NAMs) [29,30] and agonist/positive allosteric modulators (PAMs) [31,32,33,34]. Small molecules have also been reported to behave as competitive antagonists [35,36,37]. Of particular interest to this study, thiazolidinone derivatives were the first chemical molecules identified by combinatorial screening to specifically activate FSHR with no cross-reactivity with the other glycoprotein hormone receptors [38]. Thiazolinidone derivatives did not compete with radiolabeled FSH for binding to FSHR, suggesting an allosteric mode of action. Moreover, slight changes in their side chains switched FSHR-coupling from Gα_s_ to Gα_i_ [39]. However, bias factors were not determined. Compound 5 (renamed T1 thereafter) was able to induce preovulatory follicular development in immature rats but was not further pursued due to unfavorable pharmacokinetic parameters [32]. A separate class of ligands was identified through structure-activity relationship studies on substituted benzamides. Candidates capable of enhancing FSHR-mediated responses in the presence of EC_20_ of FSH, with no cross-reactivity with luteinizing hormone choriogonadotropin receptor (LHCGR) or thyroid stimulating hormone receptor (TSHR), were identified [33]. This class of ligands has not been studied yet for their ability to activate FSHR in the absence of FSH. Interestingly, we recently reported that two chemically distinct LMW agonists belonging to these two chemical classes (i.e., benzamide, termed B3, and thiazolidinone, termed T1) were capable of pharmacologically programming endosomal signaling and post-endocytic trafficking of the FSHR [40]. In the present study, we systematically explored the ability of four LMW allosteric ligands belonging to these two chemical classes (i.e., B1, B2, B3, and T1, Figure 1) to induce biased signaling at the FSHR in the absence of FSH.

## 2. Results

### 2.1. Diversity in the Recruitment of G Proteins, β-Arrestin 2, and in cAMP Production

**Figure 2:**
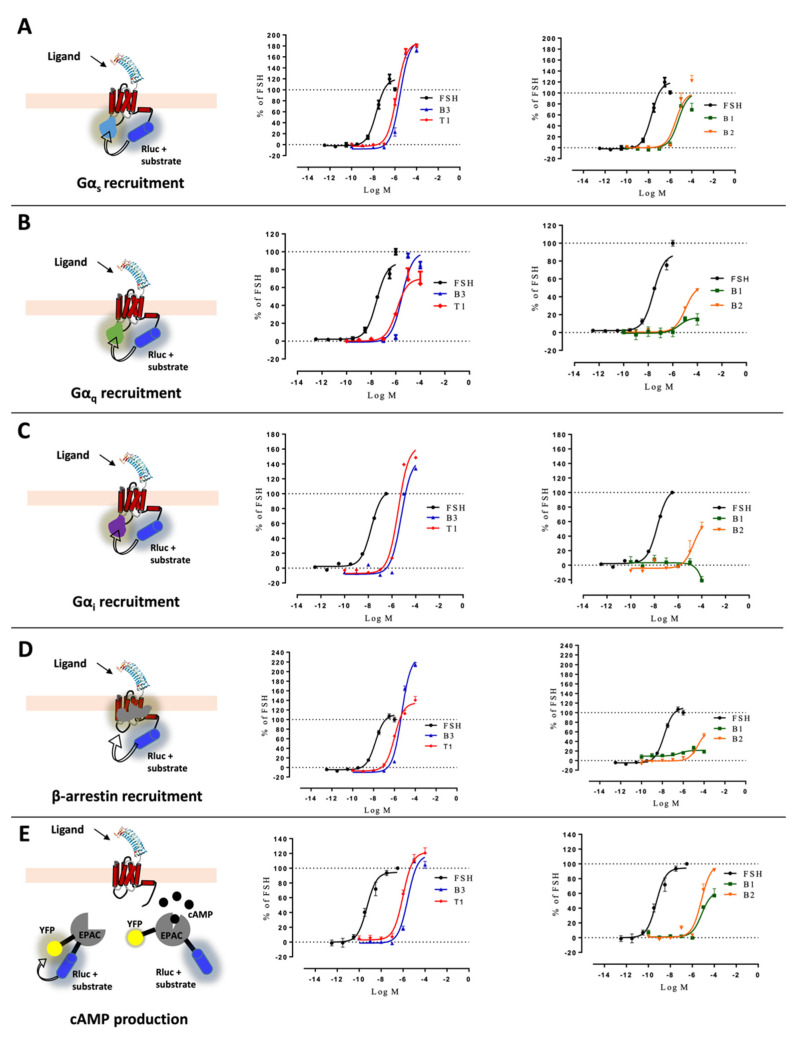
Profiling of LMW ligands vs. FSH in HEK293 cells. (**A**) Gαs recruitment (light blue), (**B**) Gαq recruitment (green), (**C**) Gαi recruitment (purple), (**D**) β-arrestin 2 recruitment (grey), and (**E**) cAMP production were measured. HEK293 cells transfected with the FSHR and the appropriate BRET sensors (Rluc stands for renilla luciferase, YFP for yellow fluorescent protein, and EPAC for exchange protein directly activated by cAMP) were challenged with increasing doses of FSH (10^−12.5^ to 10^−6.5^ M) or LMW ligands (10^−10^ to 10^−4^ M). Concentration/activity curves were measured for the different read-outs. Signals were monitored for 30 min and normalized, considering the FSH maximal response as 100%. The area under the concentration/activity curves generated by each compound was plotted and fitted using non-linear regression. Data are represented as mean ± SEM from at least six independent experiments.

We first assessed the specificity of the effects mediated by B1, B2, B3, and T1. Mock transfected HEK293 cells treated with either LMW ligand or FSH showed no cAMP response, while forskolin led to a significant increase in cAMP (Supplementary Figure S1A). In HEK293 cells expressing FSHR, cAMP response induced by either FSH or LMW ligands could be antagonized using a previously validated antagonist (T2, Figure 1; Supplementary Figure S1B–F) [39]. We next analyzed the recruitment of Gαs, Gαq, Gαi, and β-arrestin 2 to FSHR using Bioluminescence Resonance Energy Transfer (BRET). This technique allows monitoring protein-protein interaction by virtue of changes occurring in the energy transfer between a donor (Renilla luciferase) and an acceptor (fluorescent protein) when both are close enough (~10–100 angstroms) to generate a signal. Additionally, we assessed cAMP production using an intramolecular BRET sensor (both the donor and the acceptor are fused to a cAMP binding domain) that undergoes a conformational change, hence BRET change, upon cAMP binding. HEK293 cells expressing FSHR were challenged with increasing concentrations of FSH or LMW ligands over a 30 min period. Kinetics were generated for each concentration (Supplementary Figure S2), and the area under the curves was plotted as a concentration/activity sigmoid (Figure 2). We normalized each concentration/activity curve with respect to the FSH maximal response, as described in the materials and methods. As shown in Figure 2, LMW ligands displayed distinctive pharmacological profiles. Overall, small molecules were significantly less potent compared to FSH in all assays, as shown by the EC_50_ comparison (Supplementary Table S1). Interestingly, B3 and T1 displayed significantly higher E_max_ than FSH for the Gα_s_ and Gα_i_ subunit recruitment (Figure 2A,C), whereas they were both full agonists for Gαq recruitment (Figure 2B). In contrast, B1 and B2 behaved as full agonists for Gαs but as partial agonists for Gα_q_ recruitment (Figure 2A,B). For Gα_i_ recruitment, B1 was neutral with a slightly inverse agonism at the very high concentration, whereas B2 was a partial agonist (Figure 2C). Measurement of β-arrestin 2 recruitment revealed striking differences: T1 displayed full agonism, B1 and B2 behaved as weak partial agonists, whereas B3 recruited β-arrestin 2 with an almost two-fold higher E_max_ (231.8 ± 4.9) compared to FSH, qualifying as a super-agonist for β-arrestin 2 recruitment (Figure 2D). For cAMP production, no major differences in E_max_ were found compared to FSH, except for B1 displaying partial agonism (Figure 2E).

### 2.2. Differences in Kinetics That Affect Potencies and Efficacies

**Figure 3:**
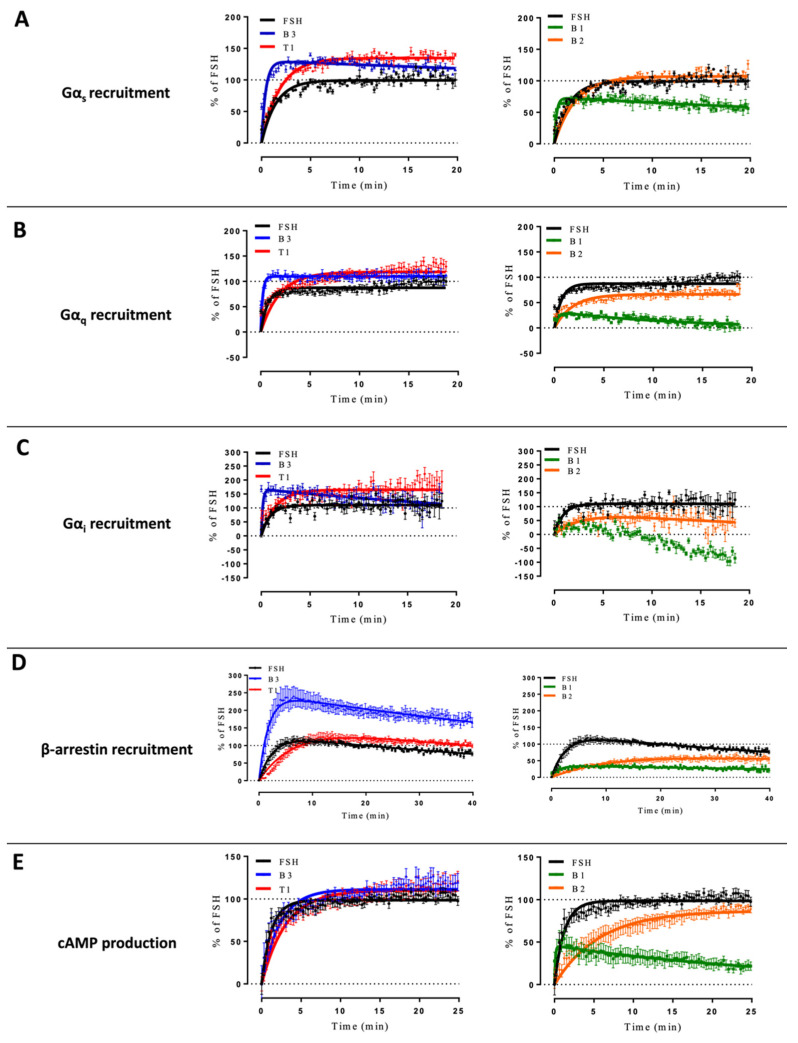
Kinetics curves induced by EC80 of FSH and LMW ligands. (**A**) Gαs recruitment, (**B**) Gαq recruitment, (**C**) Gαi recruitment, (**D**) β-arrestin 2 recruitment, and (**E**) cAMP production were measured. HEK293 cells transfected with FSHR and appropriate BRET sensors were stimulated with EC_80_ of FSH or LMW ligands. Signals were monitored for 20 to 40 min, depending on the read-out. Kinetic curves corresponding to each read-out were normalized considering the signal elicited by FSH at 20 min as 100%. Kinetics curves were plotted as a function of time. Data are represented as mean ± SEM from at least six independent experiments.

**Figure 4:**
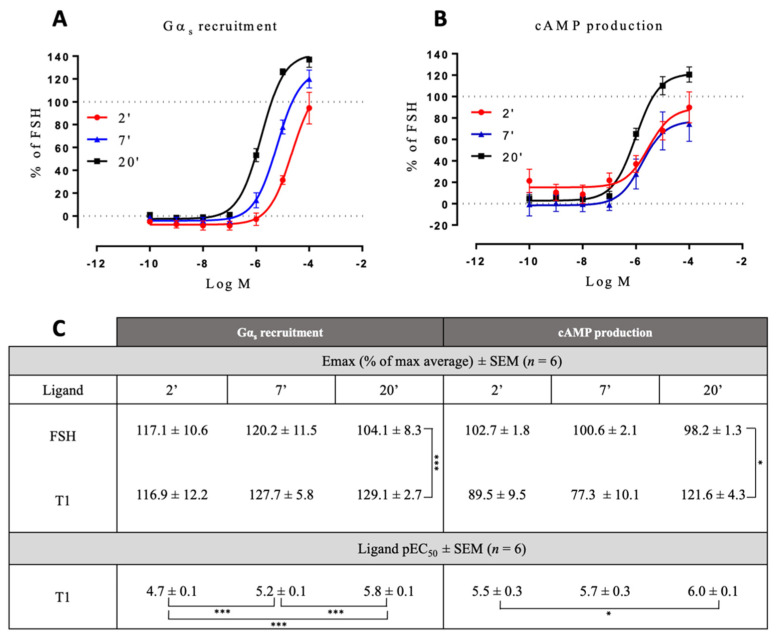
Concentration/activity curves of T1 vs. FSH at different time points. Gαs recruitment (**A**) and cAMP production (**B**) were measured at 2, 7, and 20 min of stimulation. Efficacies and potencies were compared (**C**). The area under the concentration/activity curves generated by T1 at the different endpoints considered (2, 7, and 20 min) were calculated. FSH maximal response at each endpoint was set at 100%, and T1-induced values were normalized accordingly. Normalized values were plotted and fitted using non-linear regression. Emax values were represented as the % of FSH Emax ± SEM. Potency values were represented as the positive logarithm of the ligand EC_50_ concentration ± SEM for each time considered. Statistical significance was assessed by unpaired t-test with Welch’s correction. *n* = 6, * *p* < 0.05; *** *p* < 0.001.

Comparing the kinetics across the different LMW ligands and concentrations, we observed markedly different kinetics compared to FSH (Supplementary Figure S2). Figure 3 shows time courses of G proteins and β-arrestin 2 recruitment to FSHR with each ligand used at its EC_80_ as sub-maximal concentration and normalized to FSH response at 20 min (Figure 3). We extrapolated T_1/2_ by using the general rise and fall exponential curve fitting equation as described in the materials and methods (Supplementary Tables S2 and S3). B3 led to significantly faster kinetics than FSH in recruiting Gα_s_, Gα_q_, Gα_i_, and β-arrestin 2. T1-induced kinetics were comparable to FSH for Gα_s_ and Gα_i_ recruitments but were slower for Gα_q_ and β-arrestin recruitments as well as cAMP production. B1 displayed a weaker and more transient profile with all the read-outs considered. B2 displayed significantly slower but sustained activation kinetics with all read-outs compared with FSH, except for Gα_s_. It has been reported by others that kinetics can impact apparent biases in signaling [41]. In this context, we compared the AUC (area under the curve) for each dose with endpoints at 2-, 7- and 20-min. Signals from each LMW ligand were normalized over the FSH signal taken at the same time point. Consistent with Klein Herenbrinck et al., we found that the different kinetics induced by T1 could indeed affect potency and efficacy as a function of the endpoint considered. Figure 4 demonstrates that T1-induced E_max_ and potencies were significantly different from FSH for both Gα_s_ recruitment and cAMP production as a function of the stimulation time.

### 2.3. Diversity of FSHR-Mediated ERK Phosphorylation and cAMP-Responsive Element-Dependent Transcription

**Figure 5:**
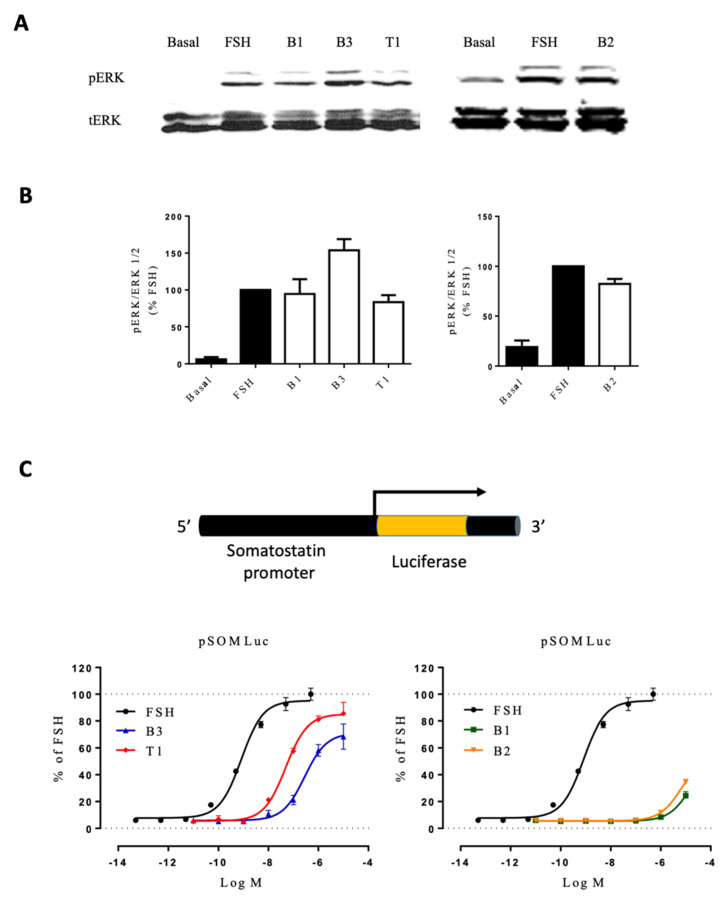
Activity of FSH vs. LMW ligands on downstream signaling. (**A**) HEK293 cells expressing the FSHR were stimulated with EC_80_ concentration of FSH or LMW ligands for 5 min. ERK phosphorylation was analyzed by western blotting. (**B**) Images were quantified and represented as normalized phosphorylated ERK/ERK1/2 values. (**C**) HEK293 co-expressing the FSHR and CRE-luciferase reporter gene were stimulated with increasing concentrations of FSH (10^−12.5^ to 10^−6.5^ M) or LMW ligands (10^−10^ to 10^−4^ M). Luciferase activities were recorded 6 h after stimulation, and signals were normalized over FSH maximal response. Values were plotted and fitted by non-linear regression. Data are represented as mean ± SEM from at least six independent experiments.

We next assessed the effect of each LMW ligand on two integrated cellular responses: (i) extracellular signal-regulated kinase (ERK1/2) phosphorylation, assessed by Western blot, and (ii) cAMP-responsive element (CRE) transcription using a reporter gene (Figure 5). ERK1/2 phosphorylation levels were evaluated after stimulating FSHR-transfected HEK293 cells for 5 min with EC_80_ of each compound (Supplementary Figures S3 and S4) and were normalized over total ERK. The 5 min time corresponded to the peak of phosphorylated ERK activation in our cellular settings, as previously described [42,43]. As shown in Figure 5, FSH and LMW ligands induced ERK phosphorylation compared to basal. Interestingly, B3 induced a higher level of ERK phosphorylation than the other LMW ligands and FSH. The Gαs/cAMP/PKA pathway is canonically activated upon FSH binding to the receptor. In particular, the second messenger cAMP binds to PKA, which, in turn, activates the cAMP response element-binding protein (CREB) in the nucleus. CREB is a transcription factor that binds to the cAMP response element (CRE) of the promoters of its target genes and leads to their activation. In this context, we were interested in analyzing the impact of LMW ligands on the FSHR-mediated Gαs/cAMP/PKA-dependent gene expression. To do this, CRE-dependent transcription was assessed after 6 h of stimulation using luciferase as a reporter gene. The data revealed striking differences compared to Gα_s_ recruitment and cAMP production. B3 and T1 were partial agonists compared to FSH, whereas B1 and B2 displayed very poor potencies, inducing weak luciferase expression only at high concentrations (Figure 5C; Supplementary Table S4).

### 2.4. Profiling in HEK293/ΔGα_s_ and HEK293/ΔARRB Cells

To further dissect the transduction mechanisms triggered by the different ligands, but also to assess the potential impact of system biases, we carried out the same profiling in HEK293 cells depleted in two key molecular effectors of FSHR, namely either Gα_s_ or β-arrestins. The levels of FSHR expression at the plasma membrane were quantified across the three cellular models by flow cytometry and were found to be similar between wild-type HEK293 and HEK293/ΔGα_s_, but slightly lower for HEK293/ΔARRB (Supplementary Figure S5).

**Figure 6:**
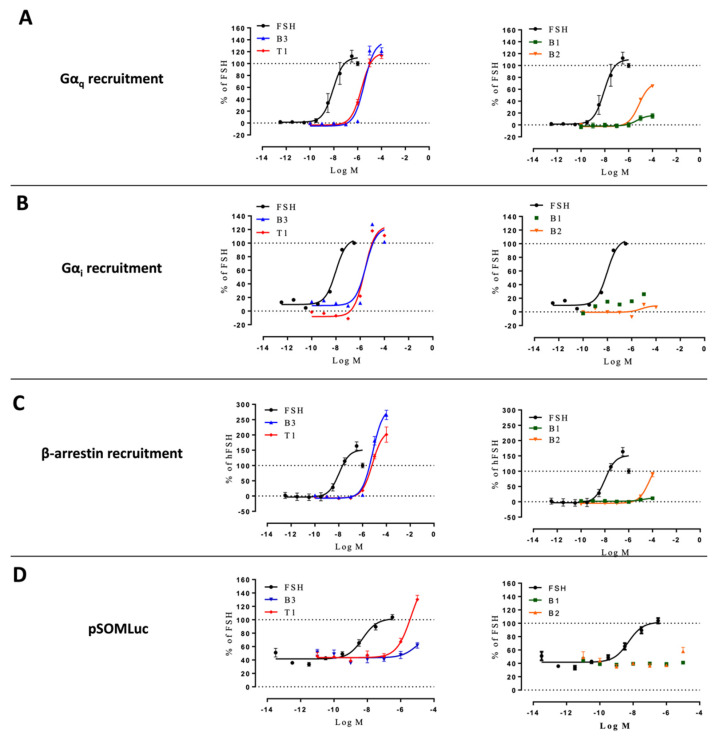
Profiling of LMW ligands vs. FSH in HEK293/ΔGαs cells. (**A**) Gαq recruitment, (**B**) Gαi recruitment, (**C**) β-arrestin 2 recruitment, and (**D**) CRE reporter gene induction were measured. Transfected HEK293/ΔGαs cells were challenged with increasing doses of FSH (10^−12.5^ to 10^−6.5^ M) or LMW ligands (10^−10^ to 10^−4^ M). Signals were monitored for 30 min. Concentration/activity curves were calculated by considering the area under the curves. Values were normalized considering FSH maximal response as 100%, plotted, and fitted using non-linear regression. Data are represented as mean ± SEM from at least six independent experiments.

Gα_q_, Gα_i_, and β-arrestin 2 recruitment, and CRE-dependent transcription were measured in HEK293/ΔGα_s_ cells stimulated with increasing doses of FSH or LMW ligands (Figure 6, Supplementary Figure S6). FSH-induced signals were arbitrarily set at 100%. The lack of Gα_s_ affected the relative efficacies of some ligands compared to FSH. In particular, B3 and T1 were less efficacious in recruiting Gα_i_ compared to the unmodified cells, while being more efficacious for Gα_q_ recruitment (Supplementary Tables S1 and S5). Interestingly, both B3 and T1 were also more efficacious than FSH in recruiting β-arrestin 2. B2 displayed a higher efficacy for Gα_q_ and β-arrestin 2 recruitments but was ineffective for Gα_i_ recruitment. Interestingly, compound B1 did not display any notable efficacy regardless of the read-out in these cells. As expected, Gα_s_ depletion strongly impaired CRE-dependent transcription, yet a clear residual activation remained and was analyzed. Compound B3 displayed weak efficacy and potency, whereas T1 induced CRE-dependent transcription with higher efficacy compared to wild-type cells. Interestingly, B1 and B2 did not trigger any measurable responses. Kinetics revealed that Gα_i_ recruitment was severely affected for all ligands and FSH compared to the wild-type cells (Supplementary Figure S6).

**Figure 7:**
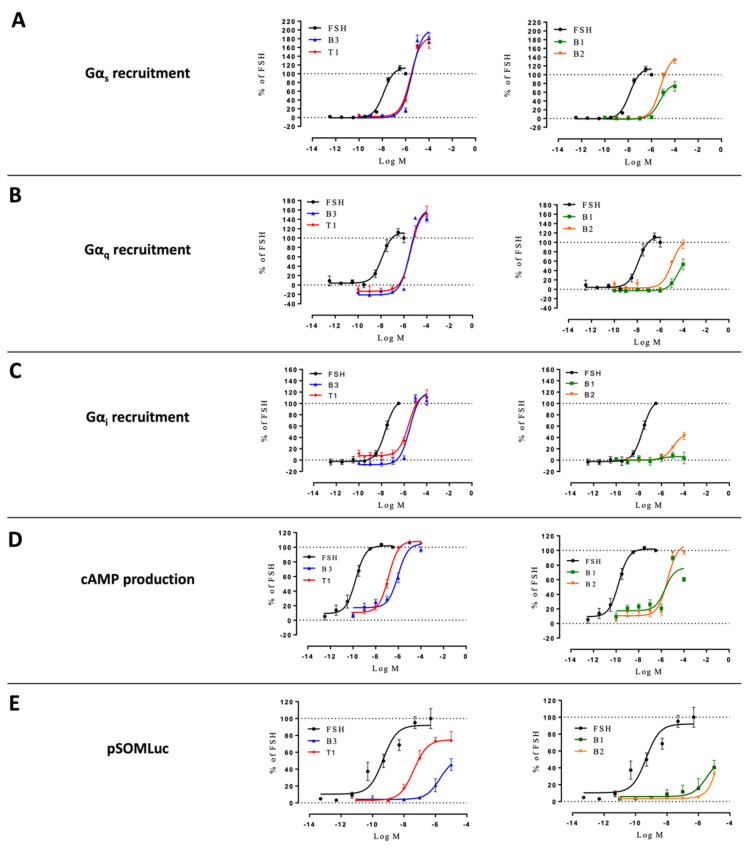
Profiling of LMW ligands vs. FSH in HEK293/ΔARRB cells. (**A**) Gαs recruitment, (**B**) Gαq recruitment, (**C**) Gαi recruitment, (**D**) cAMP production, and (**E**) CRE reporter gene induction were measured. Transfected HEK293/ΔARRB cells were challenged with increasing doses of FSH (10^−12.5^ to 10^−6.5^ M) or LMW ligands (10^−10^ to 10^−4^ M). Signals were monitored for 30 min. Concentration/activity curves were calculated by considering the area under the curves. Values were normalized considering FSH maximal response as 100%, plotted, and fitted using non-linear regression. Data are represented as mean ± SEM from at least six independent experiments.

We next investigated the effects of our ligands in a system where both β-arrestin 1 and 2 were genetically depleted (i.e., HEK293/ΔARRB). Again, FSHR-transfected cells were challenged with increasing doses of FSH or LMW ligands. G proteins, cAMP production, and CRE-luciferase expression were analyzed (Figure 7; Supplementary Table S5). B1 reached higher efficacy in Gα_s_ recruitment, while B3 and T1 showed less efficacy in recruiting Gα_i_ in HEK293/ΔARRB compared to the wild-type counterpart. However, the general overall behavior of these three LMW ligands on upstream signaling was maintained in HEK293/ΔARRB compared to wild-type HEK293. Interestingly, the lack of β-arrestin significantly affected B3-induced reporter gene expression as shown by the decreased efficacy compared to wild-type HEK293 (71.0 ± 2.7 vs. 53.4 ± 7.1, respectively), consistent with this compound being the most efficacious at inducing β-arrestin 2 recruitment in wild-type cells. The kinetics indicated that β-arrestins depletion of β-arrestins did not impair Gα_i_ recruitment the way Gα_s_ depletion did (Supplementary Figure S7).

### 2.5. Analysis of Bias Factors and Multi-Dimensional Comparison of Pharmacological Efficacies

**Figure 8:**
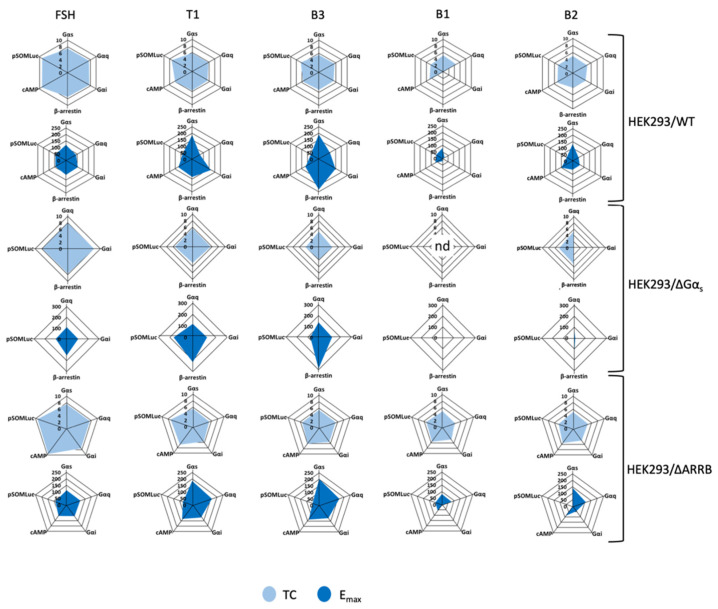
Radial graph representation of FSH and LMW ligand-induced transduction coefficients (TC) and maximal efficacies (Emax). Transduction coefficients and maximal efficacies were obtained after curve-fitting of FSH and LMW ligand-induced concentration/activity curves and are represented as radial graphs. Radius for TC is in logarithmic scale while radius for Emax is normalized, considering FSH maximal response as 100%, and is shown in linear scale. B1-induced transduction coefficients in HEK293/ΔGαs were not identifiable, and the relative radial graph was labeled as “not determined” (nd).

To visualize the pharmacological activity induced by the LMW ligands, we first graphically represented the values for the transduction coefficients (Supplementary Tables S6–S8) and the maximal efficacies (Supplementary Tables S1A and S5A,C) in radial graphs (Figure 8). Such representation visually correlates an intrinsic pharmacological parameter (i.e., the transduction coefficient) to the maximal efficacy of each ligand for the read-outs considered. As expected, FSH-induced transduction coefficients and maximal efficacies were balanced in the three cellular models considered. On the contrary, LMW ligands showed imbalance for different read-outs, highlighting a high pharmacological diversity. It is interesting to note that no B1-induced transduction coefficients in the HEK293/ΔGα_s_ cells were statistically identifiable except for the read-out Gαq; therefore, the radial plot was not determined (nd).

**Figure 9:**
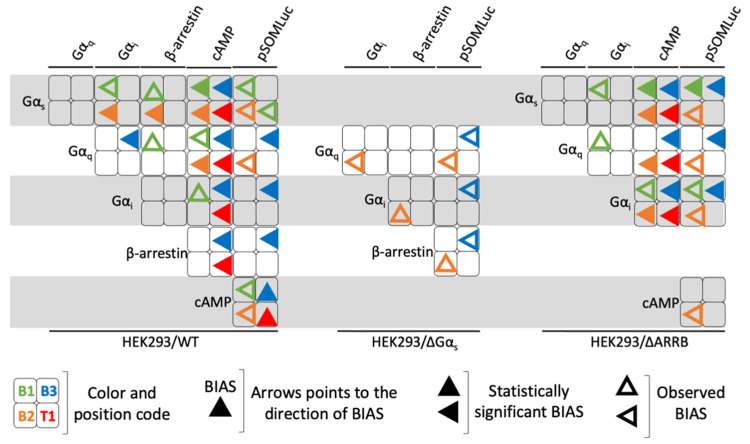
Graphic representation of the biased pharmacological behavior induced by LMW ligands. The biased pharmacological behavior of each LMW (represented in a color code, B1 = green, B2 = orange, B3 = blue and T1 = red) ligand is represented in the shape of an arrow pointing to the direction of bias for each of the pair of read-outs considered in the three cellular contexts. Statistically significant biases (full arrow) as well as observed biases (empty arrow) are shown.

We then calculated bias factors for each compound compared to the endogenous ligand FSH and for each pair of read-outs across the three cellular models, using the operational model of agonism, as described in the materials and methods (Supplementary Tables S9–S11). In Figure 9, bias values were graphically represented as arrows pointing to the direction of the bias (i.e., one read-out in each pair considered). In several cases, as described in the materials and methods, we were not able to accurately determine the bias value, reflecting a concentration-activity curve that was very weak or flat, for at least one of the two read-outs. In such cases, the fitting of the sigmoid is not robust and can lead to unreliable bias values. Since there was a clear bias towards one read-out, where we could not perform statistical analysis, we decided to represent such cases with empty arrows (observed bias) as opposed to full arrows (statistically significant bias). As shown in Figure 9, each compound displayed a unique profile, indicating pharmacological diversity. We also noticed that bias profiles varied as a function of the cell model considered, confirming the importance of the cellular context when assessing biases. Noticeably, all ligands were biased towards Gα_s_ compared to cAMP in both wild-type and HEK293/ΔARRB cells. There was also a general trend in favor of a Gα_s_-oriented bias for all ligands in HEK293/ΔARRB cells as well. It should be noted that B1 appeared as the most balanced ligand, mostly due to its low efficacy and potency in many read-outs that generated no bias values. This phenomenon is particularly visible in wild-type HEK293 and HEK293/ΔGα_s_ cells in which B1 did not display any activity except a significant bias in favor of Gα_s_ over cAMP in wild-type HEK293. Consistent with its strong dependence on Gα_s_, B1 was also largely biased in favor of Gα_s_ recruitment in HEK293/ΔARRB. Interestingly, B3 was significantly biased for β-arrestin 2 compared to cAMP and CRE-luciferase in wild-type, while T1 was significantly biased for arrestin 2, only compared to CRE-luciferase. Moreover, in wild-type cells, ligands B3 and T1 were significantly biased towards Gα_i_ compared to cAMP, while B3, B2, and T1 were significantly biased towards cAMP compared to Gα_q_. These two biases remained consistent in HEK293/ΔARRB cells.

**Figure 10:**
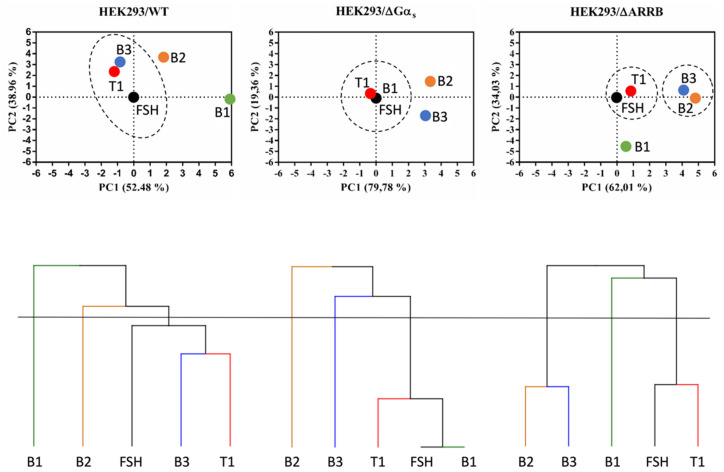
Hierarchical clustering and principal component analysis of LMW ligand-induced bias factors. Continuous lines represent the cut-off set up of a number of clusters equal to three. Clusters of ligands are highlighted with dotted lines. Note that in the PCA graph, corresponding to HEK293/ΔGαs, FSH and B1 are perfectly overlaid.

To better discriminate pharmacological efficacies of FSH and LMW ligands across the multiple dimensions in the three cellular models explored, we performed principal component analyses (PCA) using the calculated bias values. Since FSH was the reference ligand, all the values entered for FSH in the PCAs were set to 0, meaning no bias. In all three PCAs shown in Figure 10, the two principal components, PC1+PC2, accounted for 91.44 to 99.14% of the variability across the ligands, hence highlighting differences in ligand behavior. Furthermore, we performed hierarchical clustering based on the PCA results to group ligands in clusters (Figure 10). Interestingly, ligands did not group in the same clusters (dotted lines) in the different cellular models, suggesting that the gene knockouts and the associated compensatory rewiring [44] led to system biases. In wild-type HEK293, B3, FSH, and T1 were clustered together; B1, T1, and FSH were in the same cluster in HEK293/ΔGα_s_, whereas two separate clusters, composed by B3 and B2 (left cluster) and by FSH and T1 (right cluster), were found in HEK293/ΔARRB. The apparent superposition of B1 and FSH in HEK293/ΔGα_s_ was clearly misleading and is not at all indicative of pharmacological equivalence of these two ligands in this cellular system. Instead, it reflects the fact that FSH, being the reference, had no bias in any of the cellular systems (hence all values = 0), yet it clearly triggered robust responses for all read-outs measured in HEK293/ΔGαs. On the contrary, B1 displayed no activity at all in HEK293/ΔGαs, hence, no bias (all values = 0). This is an illustration of the type of caveats that can arise when running such systematic bias analyses.

## 3. Discussion

FSHR has long been a target for pharmaceutical companies since FSH is administered to patients in ovarian stimulation protocols [45]. Over the years, several attempts were made to identify suitable orally-active LMW substitutes of FSH [27,34,36,46]. However, no LMW ligand-exhibiting undisputable and well-characterized biased agonisms at the FSHR have been reported so far. In the current study, we took advantage of previously described thiazolidinone and benzamide derivatives and investigated their ability to trigger biased signaling compared to FSH [33,38]. Of note, our panel included three benzamide derivatives that presented a common core structure. We capitalized on highly-sensitive BRET sensors to carry out multiplexed measurements encompassing the main known coupling mechanisms of the FSHR: Gα_s_, Gα_q_, Gα_i_, and β-arrestin 2 recruitments [4,47]. We also assessed cAMP production, ERK phosphorylation, and CRE-dependent transcription—three downstream cellular responses known to be induced by FSH. Concentration-activity experiments were conducted for all read-outs but ERK phosphorylation to achieve quantitative comparisons (efficacy and potency) between FSH and the different ligands. Then, we applied operational model-derived analysis to the whole dataset to calculate the transduction coefficients and bias factors of all the possible pairs of read-outs.

Our data confirmed that FSH and the LMW ligands were able to recruit Gα_q_ and Gα_i_, in addition to the canonical Gα_s_ protein; thereby, supporting the existence of G protein promiscuity at the FSHR [14]. Importantly, our results reveal that the different LMW ligands lead to distinct biases at the FSHR. Indeed, B3 behaved as a super-agonist for Gα_s_ and β-arrestin 2 recruitment compared to FSH. Moreover, it was statistically biased towards β-arrestin 2 when compared to cAMP. Consistent with this finding, we observed that B3 induced more ERK phosphorylation than FSH in wild-type HEK293 cells and that its CRE-dependent transcription was more affected than the one elicited by FSH or other LMW ligands in HEK293/ΔARRB cells. T1 also showed β-arrestin 2 bias compared to cAMP and CRE-dependent transcription. Similar to B3, T1 also demonstrated two-fold higher efficacy for Gα_s_ protein recruitments while being slightly more efficacious at inducing cAMP production compared to FSH. However, its β-arrestin recruitment efficacy was significantly reduced compared to B3. In contrast, B1 displayed full agonism for Gα_s_ and partial agonism for cAMP production, respectively, in wild-type HEK293 cells; whereas, it showed little to no pharmacological activity in all other assays, including β-arrestin recruitment. In line with this Gα_s_-biased profile, B1 failed in inducing any significant response in HEK293/ΔGα_s_ cells. Compound B2, on the other hand, displayed full agonism for Gα_s_ recruitment and cAMP production but was only partial agonist for all other read-outs. These data demonstrated an unprecedented pharmacological diversity at the FSHR. These ligands represent novel molecular tools that can serve to further dissect FSHR signaling.

We noticed that LMW ligands displayed less bias towards cAMP production compared with Gα and β-arrestin recruitment. The exact mechanisms that determine the orientation of the bias remain largely unknown, even though more studies are attempting to link signaling behaviors to receptor structural conformations. In the case of cAMP production vs. Gα and β-arrestin recruitment, the stoichiometry of the elements at play is vastly different: for Gα or β-arrestin recruitment to the receptor, the stoichiometry is 1:1, meaning that one activated receptor recruits one Gα protein/β-arrestin. In contrast, cAMP production is strongly amplified: activation of one receptor leads to plenty of cAMP molecules produced, making cAMP response less sensitive to pharmacological modulation. We previously reported that only a few percent of occupied FSHRs still generate maximum cAMP responses, whereas, Gα or β-arrestin recruitment are perfectly correlated with receptor occupancy [48]. This discrepancy may explain why LMW compounds showed less bias towards cAMP production.

Importantly, distinct pharmacological profiles were obtained with three molecules that are relatively similar. Indeed, B1, B2, and B3 share about 90% of their chemical structure [33], yet their pharmacological profiles were widely different. The same is true with T1 and T2, which also share 90% of their chemical structure. However, T1 was a biased agonist, while T2 was an antagonist [39]. This observation is in line with the constantly growing number of examples of slight chemical modifications leading to profound pharmacological biases at other GPCRs.

Another interesting finding of our study is that, despite some of the LMW ligands (i.e., B3 and T1) being full- or even super-agonists for G protein recruitments, cAMP production and β-arrestin 2 recruitment compared to FSH was less efficient for CRE-dependent transcription. From this observation, we hypothesized that the different kinetic profiles elicited at the FSHR by the different ligands could account for the differences measured at the transcriptional level as well as for ligand-specific receptor assembly at the membrane. Signal compartmentalization could also play a role as it was recently shown that FSHR and LHCGR produce a large proportion of their cAMPs from endosomes [40,43]. It is therefore conceivable that LMW ligands lead to differential activations of the plasma-membrane vs. endosomal cAMP production. Indeed, increased T1-induced cAMP signaling is endosomal and, at the same time, is insensitive to the negative regulation of APPL1. Interestingly, the importance of kinetics in pharmacological efficacy at the dopamine D_1_ receptor has been recently explored [41]. In particular, the D_1_ receptor did not display the same kinetics of activation with the different ligands analyzed. Similarly, we found that concentration/activity curves made from different endpoints could lead to different relative efficacies and potencies compared to FSH. Looking at the full kinetics, we found that LMW ligands displayed different profiles that were slow, fast, or transient compared to FSH. Our study further highlights the importance of considering kinetics when pharmacologically profiling ligands.

Given the biased properties of the different LMW ligands in our panel, one can wonder what effects they would have on reproduction. For example, the selective activation of a specific FSHR-mediated signaling pathway by a biased compound may enhance the ovulation output in ART while reducing the risk for OHSS. In a similar way, such biased properties may contribute to dissect the molecular mechanism leading to reproduction-related disorders. Interestingly, T1 was already tested in immature rats. Despite good potency and efficacy in vitro, serum T1 was undetectable in vivo. Effects on ovulation were achieved only when T1 was continuously perfused with a pump. Moreover, some genotoxic effects were reported [32]. On the contrary, B1, B2, and B3 were not yet evaluated in vivo. Other benzamide derivatives from the same series were tested in immature rats. They displayed acceptable pharmacokinetic parameters but were not tested for their effects on ovulation [33]. In this context, evaluating B1, B2, and B3 in vivo could help decipher the respective contributions of G proteins and β-arrestin on reproduction, thereby assessing the potential of FSHR-targeted biased drugs for clinical applications in the field of reproductive medicine.

System bias has been identified as a potential confusion factor for pharmacological profiling of ligand bias [26]. System bias is often evoked when comparing different cell types and/or physiological contexts. Here we reasoned that the advent of CRISPR Cas9 knock-out models of HEK293 could be an interesting opportunity to look at system bias from a different perspective. Indeed, it was recently reported that such gene depletion forced an important rewiring of signaling pathways in order for the knock-out cells to adapt and compensate for the missing factor [44]. We compared, side-by-side, three models of HEK293 cells wild-type or depleted for Gα_s_ and both β-arrestin isoforms. We clearly found that our ligands did not display the same pharmacological profiles compared to FSH in the different models, further confirming the importance of system bias. We demonstrated that even a discrete alteration affecting one transduction partner could lead to pathway rewiring, completely changing the pharmacological profiles, even though the receptor was expressed at similar or slightly lower levels and the assays were identical.

In addition, we observed that Gα_i_ recruitment was profoundly reduced for all LMW ligands and for FSH in HEK293/ΔGα_s_ compared to wild-type and ΔARRB HEK293 cells. Even though the residual effects were sufficient to determine potencies and efficacies for Gα_i_ recruitment, the drastic reduction of the overall coupling to Gα_i_ in the absence of Gα_s_ suggests that a possible Gα_s_/Gα_i_ switching occurs at the FSHR. A switching from Gα_s_ to Gα_i_ coupling, subsequent to phosphorylation of β2-adrenergic by PKA, was previously reported [49,50]. This is yet another illustration of the importance and confounding nature of system bias.

In addition to the importance of kinetics and system biases, our study pointed to some limitations in the way pharmacological biases are currently statistically measured. State-of-the-art methods rely on the Black and Leff operational model of agonism to quantify bias [51,52]. The main limitation of this approach is that it is based on fitting a sigmoid to concentration/activity curves. It works robustly when actual responses can be measured. However, as expected in the case of biased ligands, some ligands do not exhibit clear efficacy in some assays. In such a case, the operational model still fits a sigmoid, but the inflection points are not reliably identified, and this generates aberrant biased values. A modification of the operational model was recently proposed to deal with such cases of extreme biases [53]. However, this approach only works for orthosteric ligands, as it relies on competition; therefore, it is not applicable to allosteric ligands. Further studies will be necessary to address this important limitation in the future and allow reliable pharmacological profiling in all situations.

Overall, we report for the first time a panel of LMW agonists presenting varied biased profiles at the FSHR. This finding will help decipher the relative contributions to the reproduction-related physiopathology of the different signaling pathways triggered by the FSHR. In that sense, our study potentially represents a significant step towards the development of improved therapeutics in reproductive medicine. Such molecules could be used to gain a better understanding of FSHR signaling, a pre-requisite to the discovery of novel FSHR-targeting drugs. In structure-activity relationship studies, these compounds may also be used to determine the chemical elements responsible for a particular drug effect.

## 4. Materials and Methods

### 4.1. Ligands and Materials

The FSHR low molecular weight ligands (B1, B2, B3, T1, T2) were provided by TocopheRx (Burlington, VT, USA). Classification and chemical structures are displayed in Figure 1. Ligands were diluted in sterile dimethyl sulfoxide (DMSO, Sigma-Aldrich, Saint-Louis, MO, USA) for conservation at −20 °C then diluted in PBS (Eurobio, Les Ulis, France) at appropriate concentrations for stimulations. Recombinant FSH was kindly donated by Merck (Darmstadt, Germany) and diluted in PBS. Ninety-six-well white microplates were acquired from Greiner Bio-one (Courtaboeuf, France). Coelenterazine H was purchased from Interchim (Montluçon, France).

### 4.2. Cell Culture and Transfection

HEK293/ΔGαs were generated by introducing null mutations into the *GNAS* gene, and the *GNAL* genes and HEK293/∆ARRB were mutated in the *ARRB1* and the *ARRB2* genes, using the CRISPR/Cas9 genome editing technology as described elsewhere [54,55,56]. Cells were cultured in a complete DMEM medium, supplemented with 10% (*v*/*v*) fetal bovine serum, 4.5 g/L glucose, 100 U/mL penicillin, 0.1 mg/mL streptomycin, and 1mM glutamine (all from Thermo Fisher Scientific, Waltham, MA, USA). Transient transfections were performed in suspension in a 96-well plate using Metafectene PRO (Biontex, München, Germany) following the manufacturer’s protocol.

### 4.3. BRET Sensors

To evaluate Gα_s_, Gα_q_, Gα_i_ recruitment, cells were transiently transfected with previously described FSHR C-terminally fused with donor Rluc8 (FSHR-Rluc8) and acceptor NES-Venus-mGs, or NES-Venus-mGsq or NES-Venus-mGsi (kindly provided by Pr. N.A. Lambert, Augusta University, Augusta, GA, USA) [47,48]. In order to measure real-time cAMP response in living cells, HEK293 cells were transiently transfected with two plasmids coding for FSHR (Tranchant et al., 2011) and the BRET-based cAMP sensor CAMYEL (kindly provided by L.I. Jiang, University of Texas, Austin, TX, USA) [57]. For the assessment of β-arrestin 2 recruitment, HEK293 cells were transiently co-transfected with plasmids coding for FSHR-Rluc8 and for β-arrestin 2 N-terminally fused to the BRET acceptor yPET (kindly provided by Dr. M.G. Scott, Cochin Institute, Paris, France) [48].

### 4.4. Cell Stimulation and BRET Measurement

Forty-eight hours after transfection, BRET measurements were immediately performed upon addition of increasing concentrations of FSH (10^−12.5^ to 10^−6.5^ M) or of LMW ligands (10^−10^ to 10^−4^ M) and 5 µM of coelenterazine H diluted in PBS. Signals were recorded for 30 min in a Mithras LB 943 plate reader (Berthold Technologies GmbH & Co., Wildbad, Germany).

### 4.5. Western Blot Analysis

Cells were stimulated with 80% effective concentrations (EC_80_) of FSH or LMW ligands for 5 min, and then the supernatants were removed. Cells were then lysed in 2X Laemmli buffer (250 mM tris-HCl (pH 6.8), 2% (*w*/*v*) SDS, 10% (*v*/*v*) glycerol, 0.01% (*w*/*v*) bromophenol blue, and 5% (*v*/*v*) β-mercaptoethanol) and equal amounts of protein were analysed by Western blot. The membranes were incubated overnight at 4 °C with anti-phospho-ERK 1/2 (P-p44/42 MAPK; Rabbit mAB; Ref:4370S; Lot 17 or Lot 23; 1:3000 or 1:1000; Cell Signalling Technologies, Danvers, MA, USA) and were reprobed with primary polyclonal antibody against total ERK2 (ERK2(C-14); sc-154; Lot #K2012; rabbit polyclonal IgG; 1:10,000; Santa Cruz Biotechnology, Dallas, TX, USA) or anti-p42/44 ERK (Cell Signalling Technology, catalog number: 9102, 1:10,000, Ref: 9107, Lot 28) followed by IRDye conjugates (IRDye800CW, 926-68071, Lot C70901-13/ IRDye680RD, 926-32211, Lot 70926-05; goat anti-rabbit; 1:300; Li-Cor Biosciences, Lincoln, NE, USA) or Horseradish Peroxidase HRP conjugated mouse anti-rabbit (Santa Cruz, sc-2357, Lot L1218, 1:10,000) as secondary antibodies. Protein bands were detected with a LI-Cor Odyssey CLX far-infrared scanner (Li-Cor Biosciences, Lincoln, NE, USA), or membranes were subsequently exposed to autoradiography films (GE Healthcare Amersham^TM^ Hyperfilm^TM^ ECL, 10534205) in a dark room. Bands were quantified by densitometry with Image Studio Ver. 2.1 (Li-Cor Biosciences, Lincoln, NE, USA).

### 4.6. Flow Cytometry

Cells (wild type HEK293, HEK293/ΔGα_s,_ and HEK293ΔARRB) were transiently transfected with either plasmid encoding for an empty pcDNA3.1 vector (mock) or N-terminally-tagged FLAG-FSHR. Cells were detached, washed, and re-suspended in working buffer (PBS w/o Ca^2+^ and Mg^2+^, 1% BSA, 2 mM EDTA) 48 h after transfection. Cells were then incubated with PE-conjugated anti-FLAG antibody (anti-DYKDDDDK-PE, Order n.: 130-101-577, Lot: 5190225429, 1:100, Milteny Biotech, Bergisch Gladbach, Germany) for 1 h at 4 °C. Cells were washed twice and re-suspended in working buffer before analysis with MACSQuant Analyzer 10 Flow cytometer (Milteny Biotech, Bergisch Gladbach, Germany). Data were analyzed and plotted with FlowJo Ver. 9.6 (FlowJo, Ashland, OR, USA).

### 4.7. Cre-Dependent Reporter Assay

HEK293 cells were transiently transfected with FSHR and the pSOM-Luc plasmid expressing the firefly luciferase reporter gene under the control of the cAMP Responsive Element of the somatostatin promoter region [58]. After 48-h transfection, cells were stimulated for 6 h with increasing doses of FSH (10^−12.5^ to 10^−6.5^ M) or LMW ligands (10^−10^ to 10^−4^ M). Luciferase activity was measured by adding Bright-Glo Luciferase assay substrate (Promega, Madison, WI, USA), and the emitted light was measured in a Mithras LB 943 plate reader. Values were expressed in relative luciferase activity units (RLU).

### 4.8. Statistical Analysis of Concentration/Activity Curves

The results are shown as mean ± SEM from at least six independent experiments. Data were analyzed and plotted using GraphPad Prism Ver.9.0 (San Diego, CA, USA). For normalizations, the values of all replicates were divided by the mean of FSH-induced maximal responses and multiplied by 100 for any given read-out. Statistical significance was assessed by an unpaired *t*-test with Welch’s correction, based on the number of datasets in each given experiment. *p*-values were considered significant when <0.05.

### 4.9. Statistical Analysis of Kinetic Curves

The results are shown as mean ± SEM from at least six independent experiments. T_1/2_ were obtained after curve fitting by using the general rise and fall exponential equation in GraphPad Prism:

*E* = *C*/(*K*_1_ − *K*_2_) × (*e*^−*K*_2_^*^t^* − *e*^−*K*_1_^*^t^*)

where T_1/2_ = log(2)/*K*_1_, as previously described by Hoare et al., 2018 [59].

### 4.10. Bias Calculation and Statistics

Transduction coefficients (R = τ/Ka) and bias factor (BF) were obtained after statistical fitting (Supplementary Figures S8–S10) of the operational model for each dose-response curve using normalized data from Figure 2, Figure 6, and Figure 7. Starting from the procedure described in van der Westhuizen et al. [60], performed in GraphPad Prism, we adapted the methodology using the D2D-software on Matlab Vers. 2016b (MathWorks, Natick, MA, USA) [61] to improve the statistical significance and avoid possible misleading procedures in data fitting due to parameter non-identifiability. For a given response and a given ligand, the corresponding concentration/activity curve was fitted using the operational model given by Equation (1):

*E* = *Basal* + (*E_max_* − *Basal*) × (*τ[L])*^*n*/((*τ[L]*)^*n* + (*Ka* + *[L]*)^*n*), (1)

where *[L]* denotes the concentration of ligand and *E* is the quantification of its effect (such as BRET ratio, for instance). The basal parameter is the baseline of the response; *E_max_* is the maximal possible response of the system; *τ* is the efficacy; *Ka* is the functional equilibrium dissociation constant of the agonist; *n* is the Hill slope of the transducer function that links occupancy to response. Equation (1) was poorly parameterized to yield proper identification of the transduction coefficient, hence using standard algebraic manipulations, Equation (1) was transformed into Equation (2):

*E* = *Basal* + (*E_max_* − *Basal*)/(1+((*[L]*/10^*LogKa+1*)/(10^*LogR* × *[L]*))^*n*), (2)

Factor *n* was set to 1 for all concentration/activity curves in order to improve parameter identifiability.

For a given pair of responses, E1 and E2, we systematically computed the bias between each LMW ligand and FSH. Equation (2) was used with *Ka* set to 1 for both responses when referring to FSH:

*E*1*_FSH* = *Basal*1 + (*E_max_*1 − *Basal*1)/(1 + (*[L]* + 1)/(10^*LogR*1 × *[L]*)), (3)

*E*2*_FSH* = *Basal*2 + (*E_max_*2 − *Basal*2)/(1 + (*[L]* + 1)/(10^*LogR*2 × *[L]*)), (4)

Then, we used Equation (2) with the same Basal and *E_max_* value, but with modified functional equilibrium dissociation constant and transduction coefficient and applied this to each LMW compound individually. Considering LMW as a generic compound belonging to the panel, equations are as follows:

*E*1*_LMW* = *Basal*1 + (*E_max_*1 − *Basal*1)/(1 + (*[L]*/10^*LogKa1_LMW* + 1)/(10^(*LogR*1 + Δ*LogR*1*_LMW*)) × *[L]*)), (5)

*E*2*_LMW* = *Basal*2 + (*E_max_*2 − *Basal*2)/(1 + (*[L]*/10^*LogKa*2*_LMW* + 1)/(10^(*LogR*2 + Δ*LogR*1*_LMW* + ΔΔ*LogBF_LMW*)) × *[L])*), (6)

Parameter Δ*LogR*1*_LMW* is the log ratio of the transduction coefficient between FSH and LMW for response E1, that is:

Δ*LogR*1*_LMW* = *LogR*1*_LMW* − *LogR*1 (7)

where ΔΔ*LogBF_LMW* is the log bias between FSH and LMW and between response E1 and E2, that is:

ΔΔ*LogBF_LMW* = Δ*LogR*2*_LMW*− Δ*LogR*1*_LMW*, (8)

Δ*LogR*2*_LMW* = *LogR*2*_LMW* − *LogR*2 (9)

Performing the fit with Equations (5) and (6) allows a better estimation of the confidence interval of the log bias ΔΔ*LogBF_LMW* as it directly enters the equation as a free parameter.

Then, we collectively fit the Equations (3) and (4) for FSH and Equations (5) and (6) for B1, B2, B3, and T1, yielding maximum likelihood estimates for all parameter values (Basal1, Emax1, R1, Basal2, Emax2, R2, Ka1_B1, Ka1_B2, Ka1_B3, Ka1_T1, Ka1_T2, Ka2_B1, Ka2_B2, Ka2_B3, Ka2_T1, Ka2_T2, ΔLogR1_B1, ΔLogR1_B2, ΔLogR1_B3, ΔLogR1_T1, ΔLogR1_T2, ΔΔLogBF_B1, ΔΔLogBF_B2, ΔΔLogBF_B3, ΔΔLogBF_T1, ΔΔLogBF_T2) in a single global fit (1000 multi-start deterministic optimization with default setting performed in D2D). For each response, we choose an additive Gaussian noise with unknown variance as the error model.

Since multi-start deterministic optimization requires a priori bounds for each parameter, we used the interval (−5, +5) for each log bias parameter value.

The 95% parameter confidence intervals for log bias values (ΔΔLogBF_B1, ΔΔLogBF_B2, ΔΔLogBF_B3, ΔΔLogBF_T1, ΔΔLogBF_T2) were determined using the profile likelihood method [62]. This approach is considered more robust than the asymptotic approach and considers finite sample size as well as parameter non-identifiability due to possible parameter correlations.

In order to test the statistical significance of a non-zero value for the log bias, we used the following likelihood ratio test for each compound: we defined the null model associated with LMW ligands as the very same model associated with Equations (3) and (4) for FSH and Equations (5) and (6) for each LMW allosteric ligand, except that ΔΔLogBF_LMW was set to 0. The maximum likelihood was found for the null model using the same optimization procedure as above (1000 multi-start deterministic optimization with default setting in D2D). Then, we used the likelihood ratio test to compare the negative log ratio of the likelihood of the null model over the likelihood of the full model with a chi-square distribution with one degree of freedom. Thus, we obtained the associated *p*-value for the significance of a non-zero log bias value vs. a zero-log bias value, taking into account possible parameter compensations when the log bias is fixed to zero. We performed this procedure for each compound individually. Importantly, these procedures (global data fitting, profile likelihood estimate, and likelihood ratio test) were performed for every pair of responses in each cell model.

### 4.11. Dealing with Parameter Non-Identifiability in Bias Calculations

Interpretation of systematic bias calculations requires caution. In particular, a number of concentration/activity curves have a flat profile for which precise estimation of the transduction coefficient is impossible, yielding very large or infinite confidence intervals. Several possibilities were considered based on visual inspection of concentration/activity curves and corresponding data fitting of the operational model (Equations (3) and (4) for FSH and Equations (5) and (6) for LMW ligands).

### 4.12. Principal Component Analysis (PCA) and Hierarchical Clustering

To analyze the high dimensional results obtained with the bias calculations, we performed a standard Principal Component Analysis (PCA) using the R package “FactoMineR” (Ver. 3.6.2, R Foundation for Statistical Computing, Vienna, Austria). Hierarchical clustering was performed based on the PCA results [63] using the HCPC function in the R package “FactoMineR” as well as the fviz_dend function in the R package “factoextra”.

## Data Availability

Not applicable.

## Supplementary Materials

The following are available online at https://www.mdpi.com/article/10.3390/ijms22189850/s1.
