# Supplementary material for: Pharmacological Characterization of Low Molecular Weight Biased Agonists at the Follicle Stimulating Hormone Receptor
Source: Int J Mol Sci. 2021 Sep 12;22(18):9850. doi: 10.3390/ijms22189850 (PMC8469697; doi:10.3390/ijms22189850)
Supplement: Supplementary file 1 [file ijms-22-09850-s001.zip › Supplementary figure legends_De Pascali et al..pdf]

Supplementary figure legend

Suppl. Fig.1*: Assessment of LMW ligands' specificity for FSHR*. (A) HEK293 cells transfected only with BRET-based cAMP sensor CAMYEL were stimulated with EC_{80} of FSH or LMW ligands. Forskolin (FSK, 10 µM) was used as positive control. Values were reported as % of FSK response. (B-F) HEK293 cells co-expressing the FSHR and the CAMYEL biosensor were treated with increasing concentrations of FSHR antagonist T2 (10^{-10} to 10^{-4} M) for 30 min at 37°C. Then, cells were challenged with EC_{80} of FSH (B) or of LMW ligands (C-F) for another 30 min. Values were represented as % of maximal responses. C/A curves were generated using non-linear regression fitting.

Suppl. Tab.1: ***Comparison of efficacies (A) and potencies (B) of FSH and ligands in HEK293 cells.*** $G\alpha_s$ recruitment, $G\alpha_q$ recruitment, $G\alpha_i$ recruitment, β-arrestin recruitment and cAMP production data from Fig.2 were analysed in GraphPad Prism. Values of E_{max} were represented as % of FSH-induced maximal response ± SEM (n=6). Potency values were represented as the positive logarithm of the ligand EC_{50} concentration ± SEM (n=6). Statistical significance were assessed by unpaired t-test with Welch's correction, *p < 0.05; **p < 0.01; ***p < 0.001.

Suppl. Tab.2: ***Comparison of kinetic $T_{1/2}$ of FSH and ligands in HEK293 cells.*** Kinetic curves from Fig.3 were subjected to curve fitting by using the general rise and fall exponential equation in GraphPad Prism. Values of $T_{1/2}$ were extrapolated and represented as value ± SEM (n=6). Statistical significance was assessed by unpaired t-test with Welch's correction, *p < 0.05; **p < 0.01; ***p < 0.001.

Suppl. Tab.3: ***Kinetic parameters extrapolated from compound kinetic analysis.*** Kinetic parameters from the general rise and fall exponential equation in GraphPad Prism for compound kinetic curve fitting are presented. A) Values of fitted parameter C. B) Values for rate constant K1. C) Value for rate constant K2. All parameters are represented as value ± SEM (n=6).

Suppl. Fig.2: ***Concentrations/kinetics curves induced by FSH and LMW ligands in HEK293 cells.*** (A) $G\alpha_s$ recruitment, (B) $G\alpha_q$ recruitment, (C) $G\alpha_i$ recruitment, (D) β-arrestin 2 recruitment and (E) cAMP production were measured. Cells co-expressing the FSHR and the appropriate BRET sensors were stimulated with increasing concentrations of FSH ($10^{-12.5}$ to $10^{-6.5}$ M) or LMW ligands (10^{-10} to 10^{-4} M). Signals

were monitored for 30 min. Kinetics curves, represented as induced BRET, were generated by subtracting at each time point the signal of the cells stimulated with PBS. Kinetics curves were plotted as function of the time.

Suppl. Fig.3: ***Analysis of FSH, B1, B3 and T1-induced ERK phosphorylation.*** Transiently transfected wild type HEK293 with FSHR were stimulated for 15 min with EC_{80} of FSH, B1, B3, or T1 and ERK phosphorylation was detected by western Blotting. Total ERK was revealed after membrane stripping for phosphorylated ERK signal normalization. Here are shown unmodified and uncropped images from three independent experiments.

Suppl. Fig.4: ***Analysis of FSH, and B2-induced ERK phosphorylation.*** Transiently transfected wild type HEK293 with FSHR were stimulated for 15 min with EC_{80} of FSH or B2, and ERK phosphorylation was detected by Western blotting. Total ERK was revealed after membrane stripping for phosphorylated ERK signal normalization. Here are shown unmodified and uncropped images from three independent experiments.

Suppl. Tab.4: ***Comparison of efficacies (A) and potencies (B) for CRE-dependent transcription in HEK293 cells.*** pSOMLuc data from Fig.5C were analysed in GraphPad Prism. Values of E_{max} were represented as % of FSH-induced maximal response ± SEM. Potency values are represented as the positive logarithm of the ligand EC_{50} concentration ± SEM (n=9). Statistical significance were assessed by unpaired t-test with Welch's correction, ***p < 0.001.

Suppl. Fig.5: ***Flow cytometry analysis of plasma membrane FSHR expression levels in the different cellular models.*** (A) wild type HEK293, (B) HEK293/ΔGαs or (C) HEK293/ΔARRB cells were transfected either with mock vector or FLAG-FSHR. Then, cells were stained with anti-FLAG-PE antibody for detection of FSHR-FLAG and analysed by flow cytometry as described in Materials and Methods. Dot-plots show side-scatter *versus* PE-intensity. Q1 represents the percentage of unstained cells and Q2 the percentage of stained cells for each dot-plot.

Suppl. Fig.6: ***Concentrations/kinetics curves induced by FSH and LMW ligands in HEK293/ΔGα$_s$ cells.*** (A) Gα$_q$ recruitment, (B) Gα$_i$ recruitment and (C) β-arrestin 2 recruitment were measured. Cells expressing the FSHR and the appropriate BRET sensors were stimulated with increasing concentrations of FSH ($10^{-12.5}$ to $10^{-6.5}$ M) or LMW ligands (10^{-10} to 10^{-4} M). Signals were monitored for 30 min. Kinetics curves,

represented as induced BRET, were generated by subtracting at each time point the signal of the cells stimulated with PBS. Kinetics curves were plotted as function of the time.

Suppl. Fig.7: ***Concentrations/kinetics curves induced by FSH and LMW ligands in HEK293/ΔARRB cells.*** (A) $G\alpha_s$ recruitment, (B) $G\alpha_q$ recruitment (C) $G\alpha_i$ recruitment and (D) cAMP production were measured. Cells co-expressing the FSHR and the appropriate BRET sensors were stimulated with increasing concentrations of FSH ($10^{-12.5}$ to $10^{-6.5}$ M) or LMW ligands (10^{-10} to 10^{-4} M). Signals were monitored for 30 min. Kinetics curves, represented as induced BRET, were generated by subtracting at each time point the signal of the cells stimulated with PBS. Kinetics curves were plotted as function of the time.

Suppl. Tab.5: ***Comparison of efficacies (A) and potencies (B) of FSH and ligands in HEK293/ΔGαs (A,B) and HEK293/ΔARRB (C,D) cells.*** $G\alpha_q$ recruitment, $G\alpha_i$ recruitment, β-arrestin recruitment and pSOMLuc data from Fig.6 were analysed in GraphPad Prism. Values of E_{max} were represented as % of FSH-induced maximal response ± SEM (n=6). Potency values were represented as the positive logarithm of the ligand EC_{50} concentration ± SEM (n=6 for BRET assays, n=9 for pSOMLuc). Statistical significance was assessed by unpaired t-test with Welch's correction, $*p < 0.05$; $**p < 0.01$; $***p < 0.001$.

Suppl. Fig.5: ***Curve fitting used for bias calculation in wild type HEK293 cells.*** Graphs represent the curve fitting of FSH and LMW ligands used to extrapolate the transduction coefficients and bias factors for each read-out considered, as explained in the Materials and Methods. Experimental data-points are also shown.

Suppl. Fig.6: ***Curve fitting used for bias calculation in HEK293/ΔGαs cells.*** Graphs represent the curve fitting of FSH and LMW ligands used to extrapolate the transduction coefficients and bias factors for each read-out considered, as explained in the Materials and Methods. Experimental data-points are also shown.

Suppl. Fig.7: ***Curve fitting used for bias calculation in HEK293/ΔARRB cells.*** Graphs represent the curve fitting of FSH and LMW ligands used to extrapolate the transduction coefficients and bias factors for each read-out considered, as explained in the Materials and Methods. Experimental data-points are also shown.

Suppl. Tab.6: ***Parameters values and lower/higher bounds of confidence intervals for the operational model in wild type HEK293.*** *The operational model is used for bias calculations,* and the values reported in the table with lower/higher bounds were calculated in a global fitting manner as described in Materials and Methods (“*Bias Calculation and statistics”)*. All parameter values are reported in log10 scale. LMW compound-induced transduction coefficients are reported as ∆logR=*LogR_LMW - LogR_FSH,* where R_FSH corresponds to FSH-induced transduction coefficients for each read-out R. As detailed in material methods, parameters Basal, Emax, n and Std (unknown standard deviation parameter) are response specific and have shared values among each ligand. The hill coefficient n is fixed to 1. The transduction coefficient (TC) and the functional equilibrium dissociation constant Ka are ligand and response dependent. For the latter, we fixed for the functional equilibrium dissociation constant *LogKa* equal to zero for the full agonist for each response. Those one are indicated on the table by the mention “fixed” in lower/upper bounds columns.

Suppl. Tab.7: ***Parameters values and lower/higher bounds of confidence intervals for the operational model in HEK293/ΔGαs.*** *The operational model is used for Bias calculations,* and the values reported in the table with lower/higher bounds were calculated in a global fitting manner as described in Materials and Methods (“*Bias Calculation and statistics”)*. All parameter values are reported in log10 scale. LMW compound-induced transduction coefficients are reported as ∆logR=*LogR_LMW - LogR_FSH,* where R_FSH corresponds to FSH-induced transduction coefficients for each read-out R. As detailed in material methods, parameters Basal, Emax, n and Std (unknown standard deviation parameter) are response specific and have shared values among each ligand. The hill coefficient n is fixed to 1. The transduction coefficient (TC) and the functional equilibrium dissociation constant Ka are ligand and response dependent. For the latter, we fixed for the functional equilibrium dissociation constant *LogKa* equal to zero for the full agonist for each response. Those one are indicated on the table by the mention “fixed” in lower/upper bounds columns.

Suppl. Tab.8: ***Parameters values and lower/higher bounds of confidence intervals for the operational model in HEK293/ΔARRB.*** *The operational model is used for Bias calculations,* and the values reported in the table with lower/higher bounds were calculated in a global fitting manner as described in Materials and

Methods (“*Bias Calculation and statistics”)*. All parameter values are reported in log10 scale. LMW compound-induced transduction coefficients are reported as ∆logR=*LogR_LMW - LogR_FSH,* where R_FSH corresponds to FSH-induced transduction coefficients for each read-out R. As detailed in material methods, parameters Basal, Emax, n and Std (unknown standard deviation parameter) are response specific and have shared values among each ligand. The hill coefficient n is fixed to 1. The transduction coefficient (TC) and the functional equilibrium dissociation constant Ka are ligand and response dependent. For the latter, we fixed for the functional equilibrium dissociation constant *LogKa* equal to zero for the full agonist for each response. Those one are indicated on the table by the mention “fixed” in lower/upper bounds columns.

Suppl. Tab.9: ***Bias factors, p values and lower/higher bounds of confidence intervals for each of the read-out pairs elicited by LMW ligands in wild type HEK293 cells.*** Values reported in the table for bias factors, p values and lower/higher bounds were calculated as described in Materials and Methods.

Suppl. Tab.10: ***Bias factors, p values and lower/higher bounds of confidence intervals for each of the read-out pairs elicited by LMW ligands in HEK293/ΔGαs cells.*** Values reported in the table for bias factors, p values and lower/higher bounds were calculated as described in Materials and Methods.

Suppl. Tab.11: ***Bias factors, p values and lower/higher bounds of confidence intervals for each of the read-out pairs elicited by LMW ligands in HEK293/ΔARRB cells.*** Values reported in the table for bias factors, p values and lower/higher bounds were calculated as described in Materials and Methods.
