# Supplementary figures and images for: Pharmacological Characterization of Low Molecular Weight Biased Agonists at the Follicle Stimulating Hormone Receptor
Source: Int J Mol Sci. 2021 Sep 12;22(18):9850. doi: 10.3390/ijms22189850 (PMC8469697; doi:10.3390/ijms22189850)

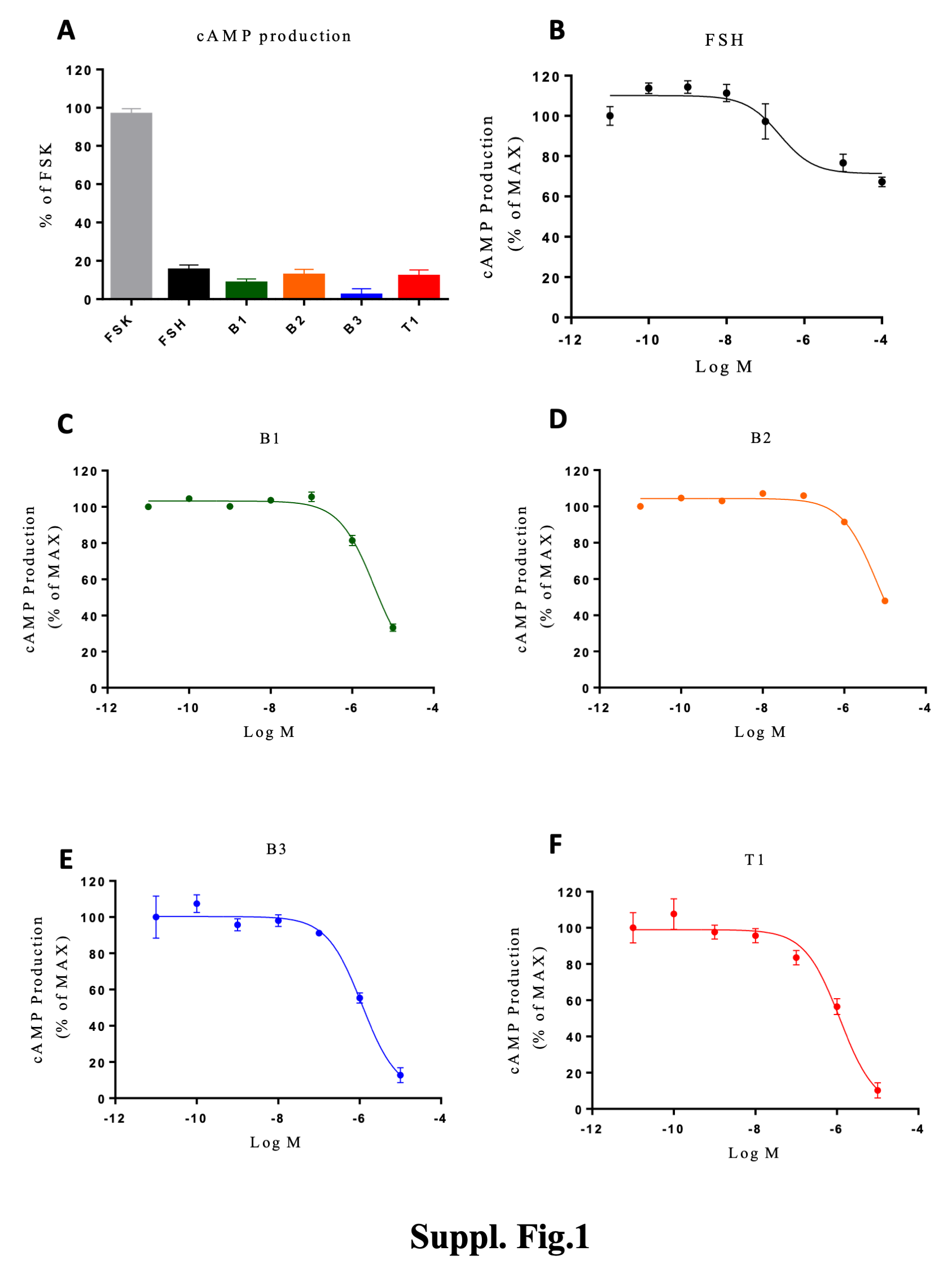

Supplement: Supplementary file 1 [file ijms-22-09850-s001.zip › Supplementary figures_De Pascali et al_/Suppl. Fig. 1.tiff]

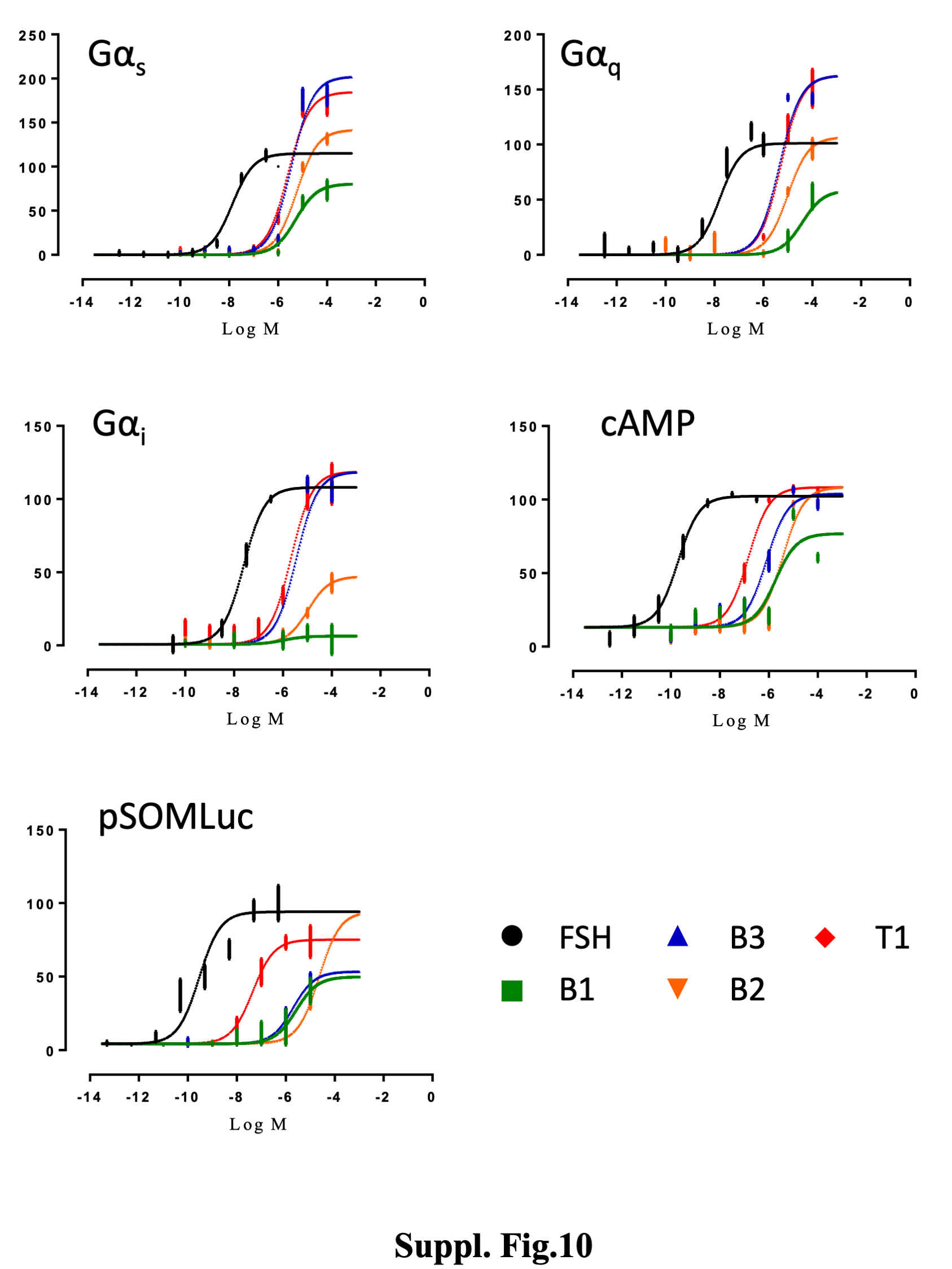

Supplement: Supplementary file 1 [file ijms-22-09850-s001.zip › Supplementary figures_De Pascali et al_/Suppl. Fig. 10.tiff]

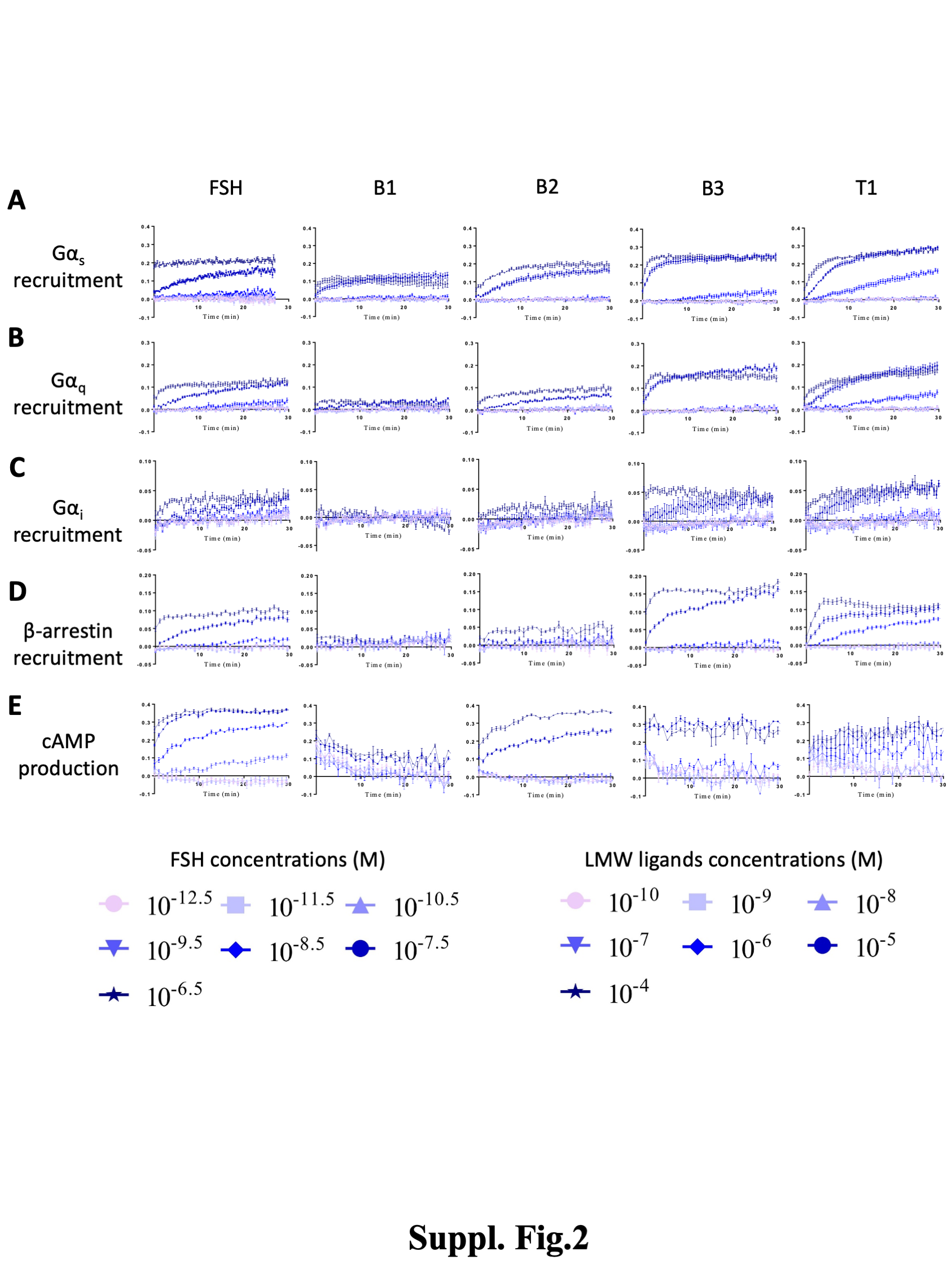

Supplement: Supplementary file 1 [file ijms-22-09850-s001.zip › Supplementary figures_De Pascali et al_/Suppl. Fig. 2.tiff]

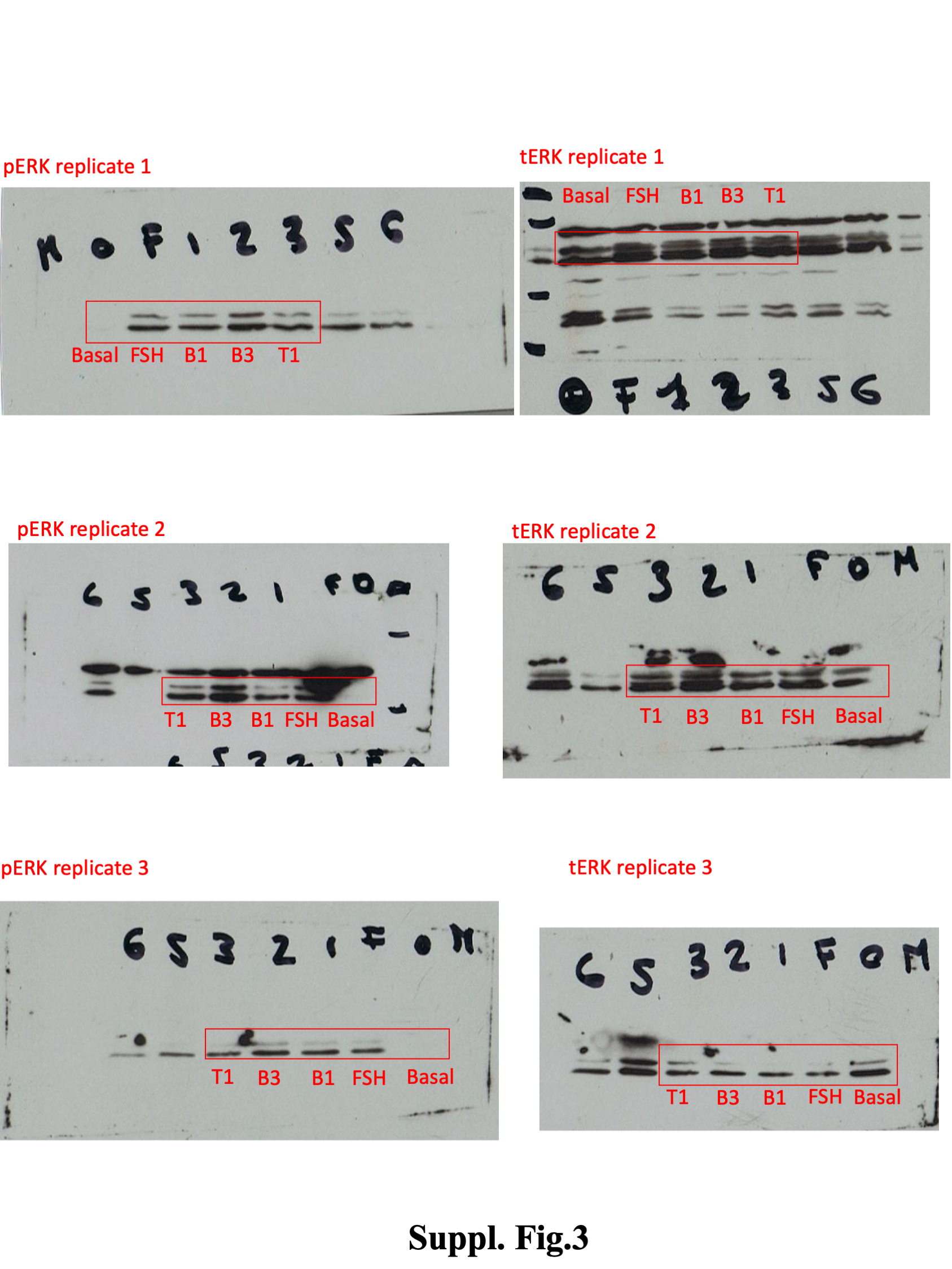

Supplement: Supplementary file 1 [file ijms-22-09850-s001.zip › Supplementary figures_De Pascali et al_/Suppl. Fig. 3.tiff]

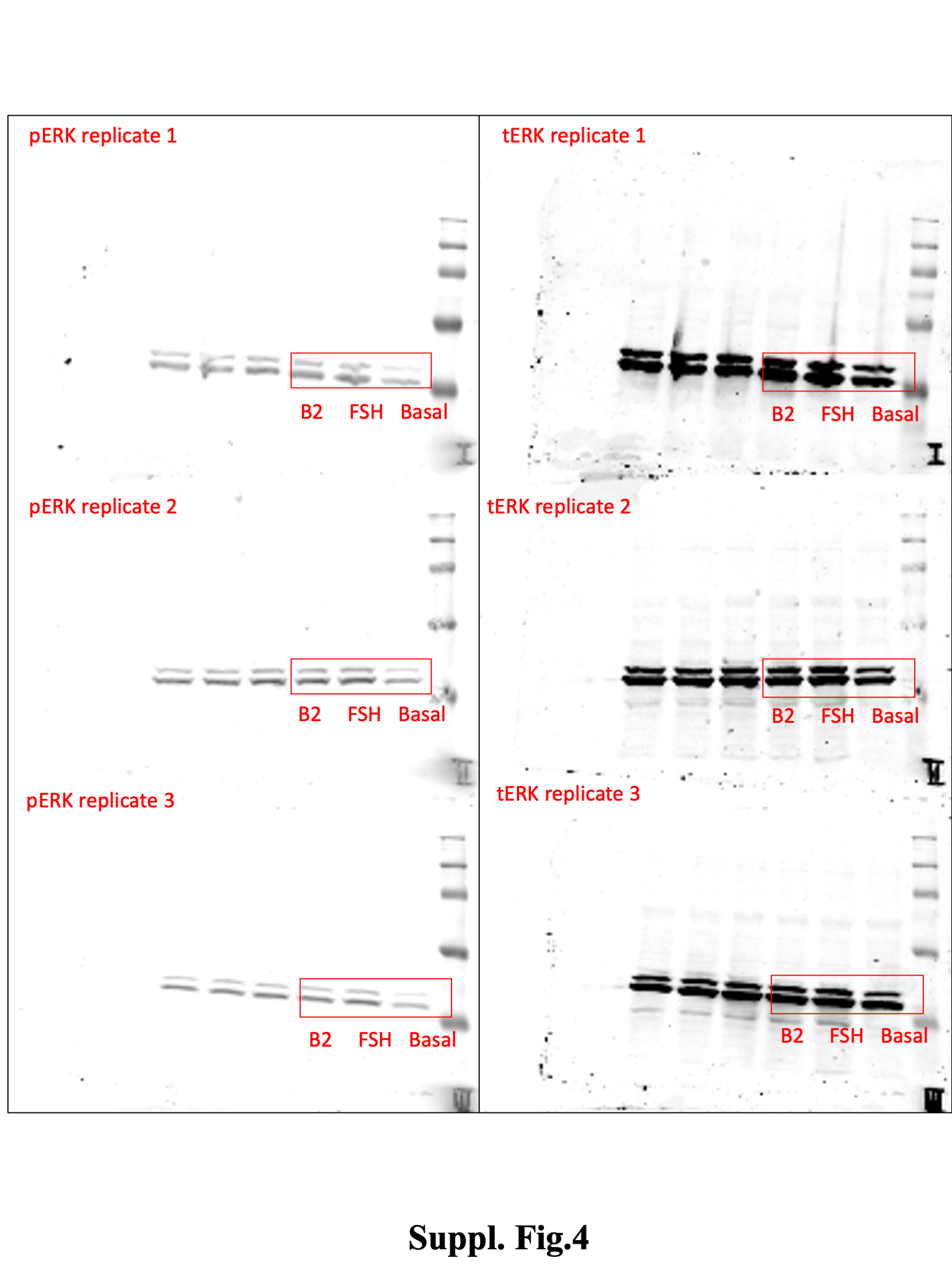

Supplement: Supplementary file 1 [file ijms-22-09850-s001.zip › Supplementary figures_De Pascali et al_/Suppl. Fig. 4.tiff]

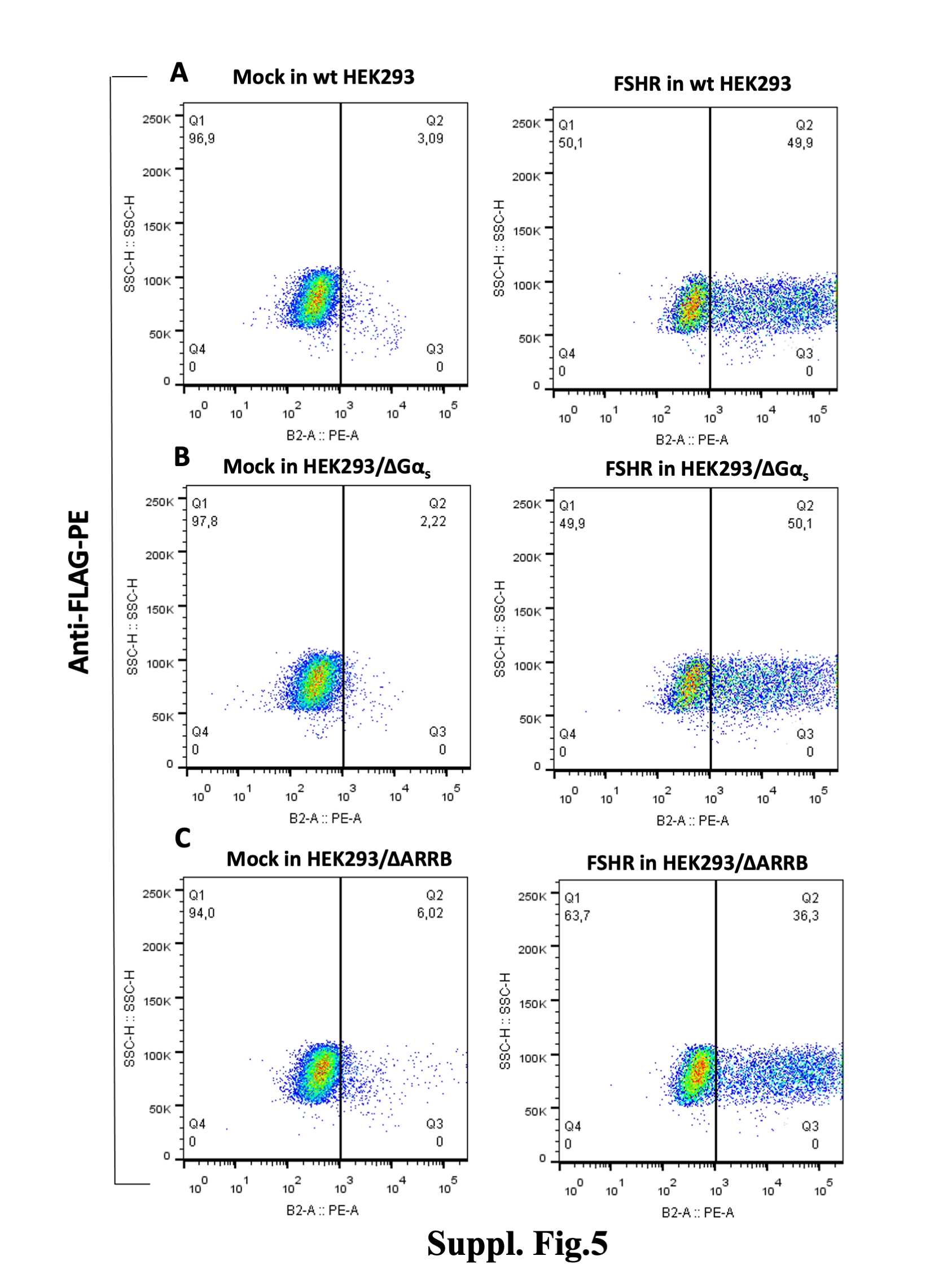

Supplement: Supplementary file 1 [file ijms-22-09850-s001.zip › Supplementary figures_De Pascali et al_/Suppl. Fig. 5.tiff]

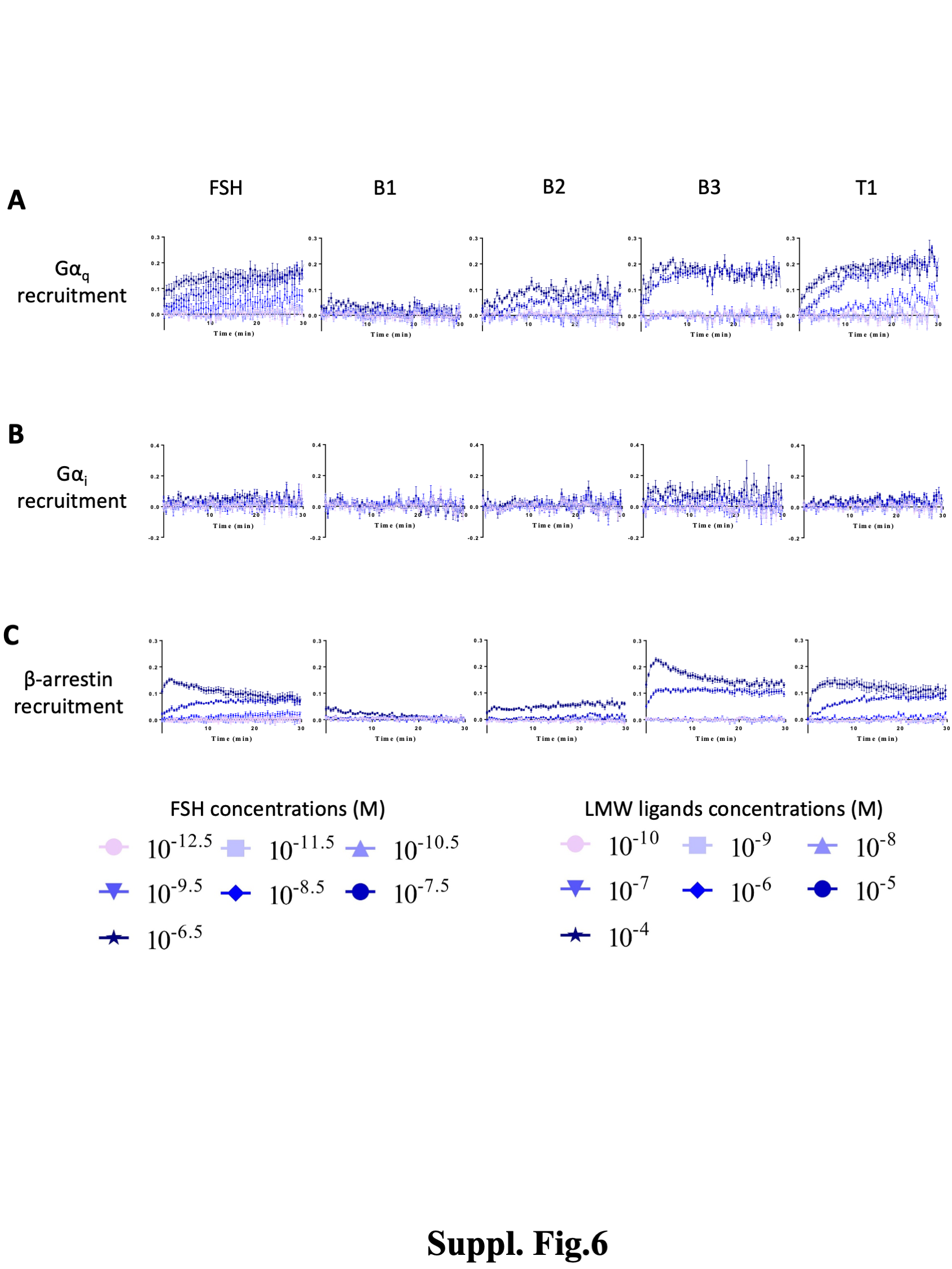

Supplement: Supplementary file 1 [file ijms-22-09850-s001.zip › Supplementary figures_De Pascali et al_/Suppl. Fig. 6.tiff]

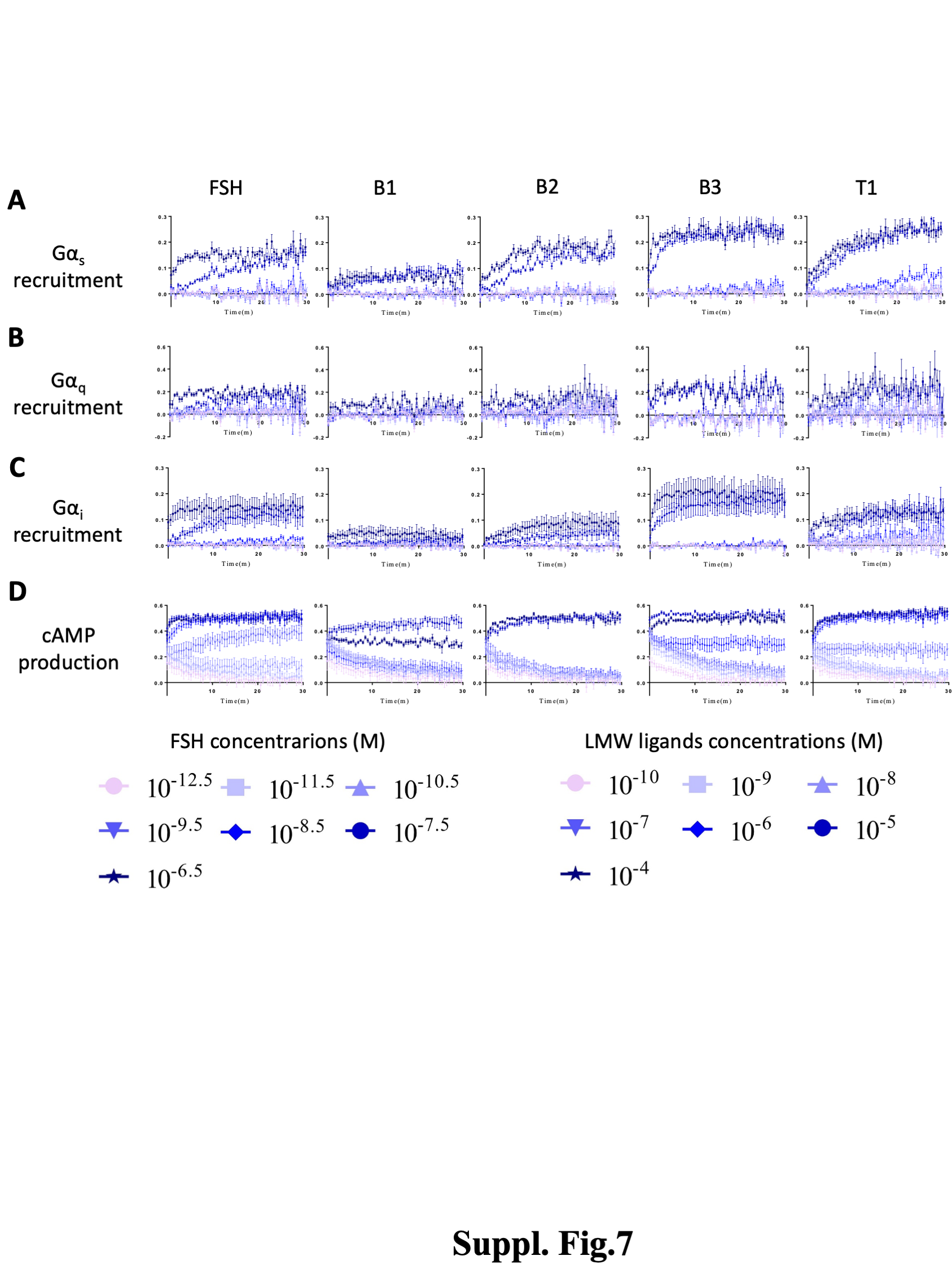

Supplement: Supplementary file 1 [file ijms-22-09850-s001.zip › Supplementary figures_De Pascali et al_/Suppl. Fig. 7.tiff]

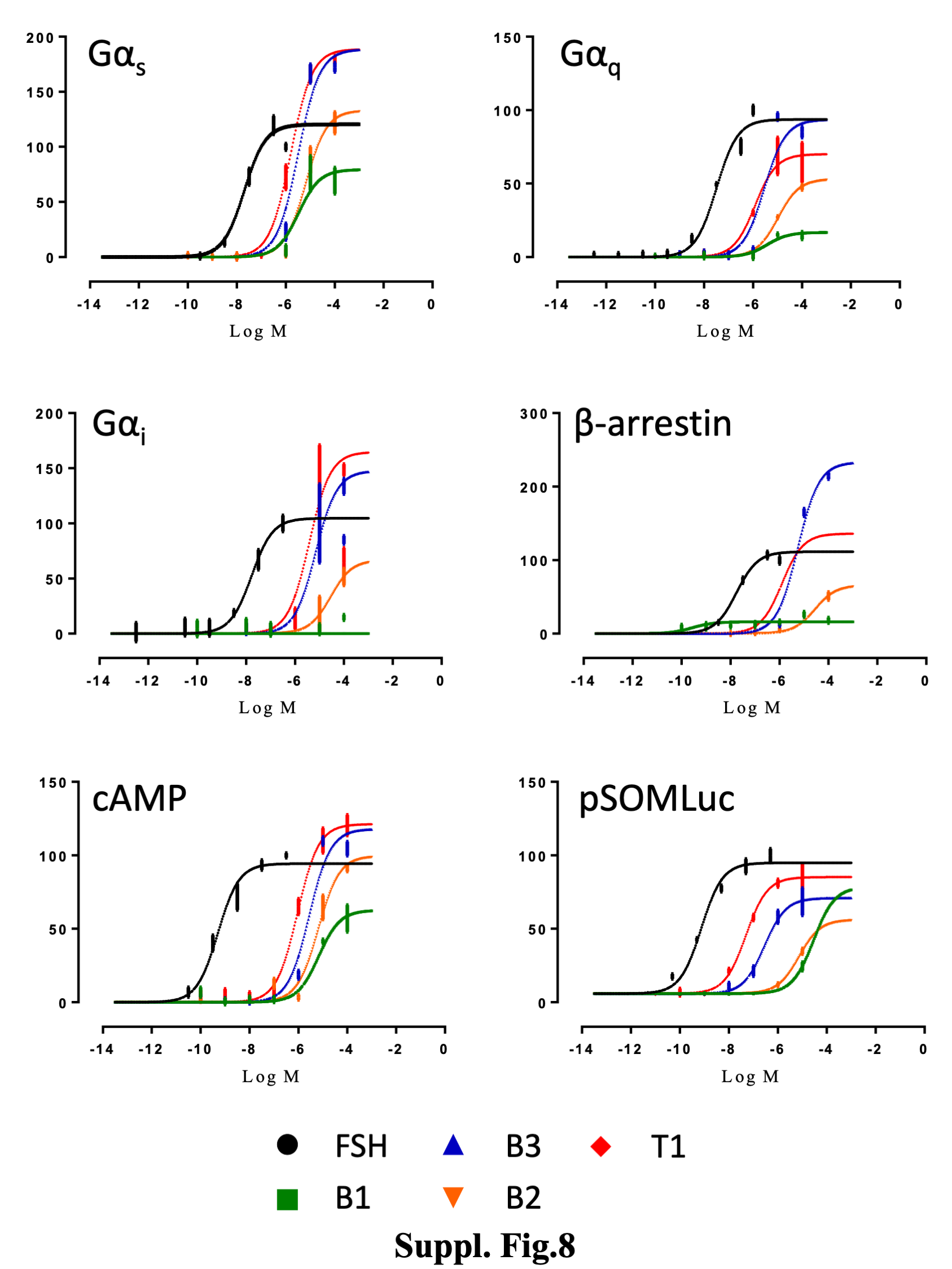

Supplement: Supplementary file 1 [file ijms-22-09850-s001.zip › Supplementary figures_De Pascali et al_/Suppl. Fig. 8.tiff]

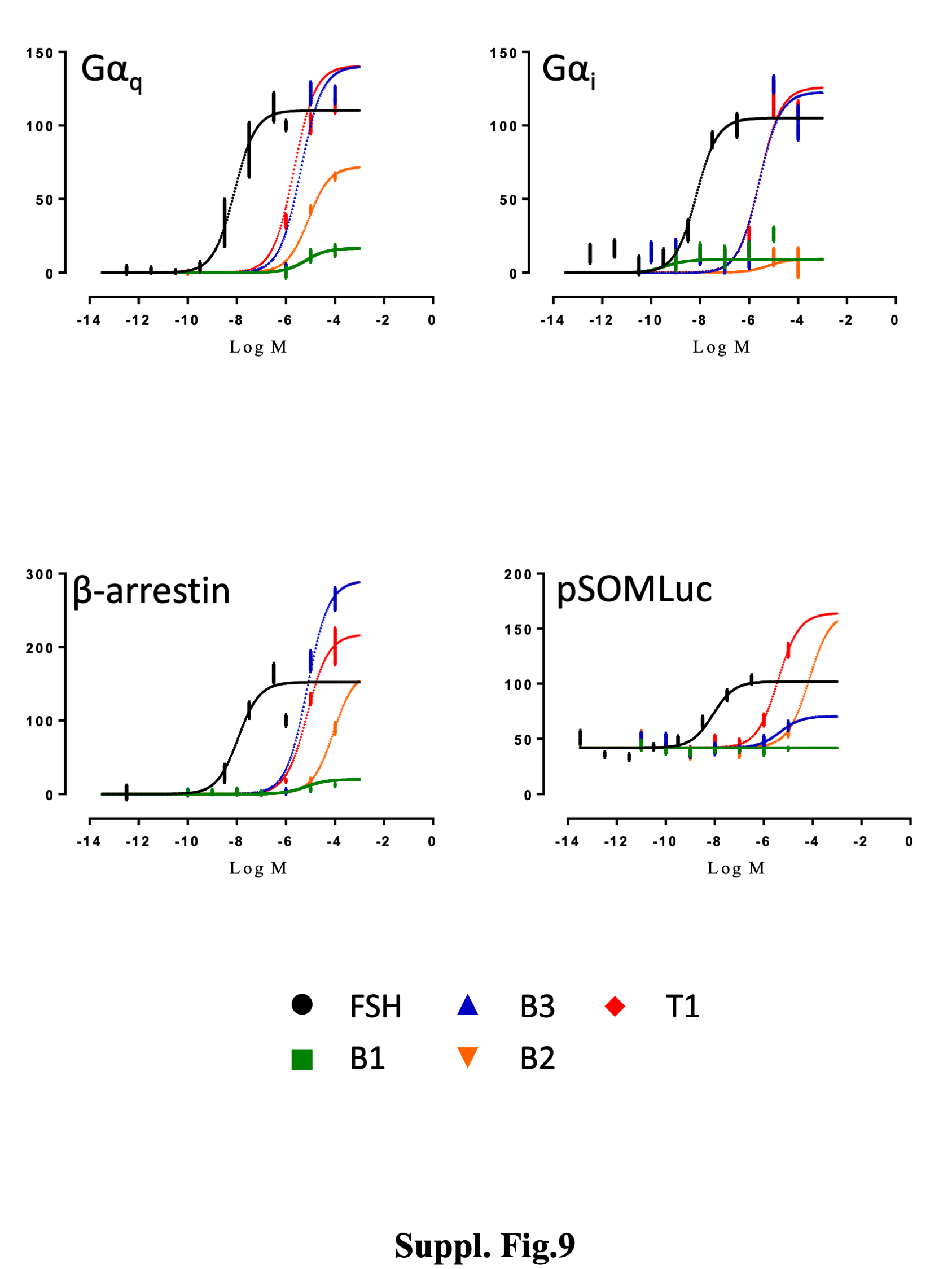

Supplement: Supplementary file 1 [file ijms-22-09850-s001.zip › Supplementary figures_De Pascali et al_/Suppl. Fig. 9.tiff]

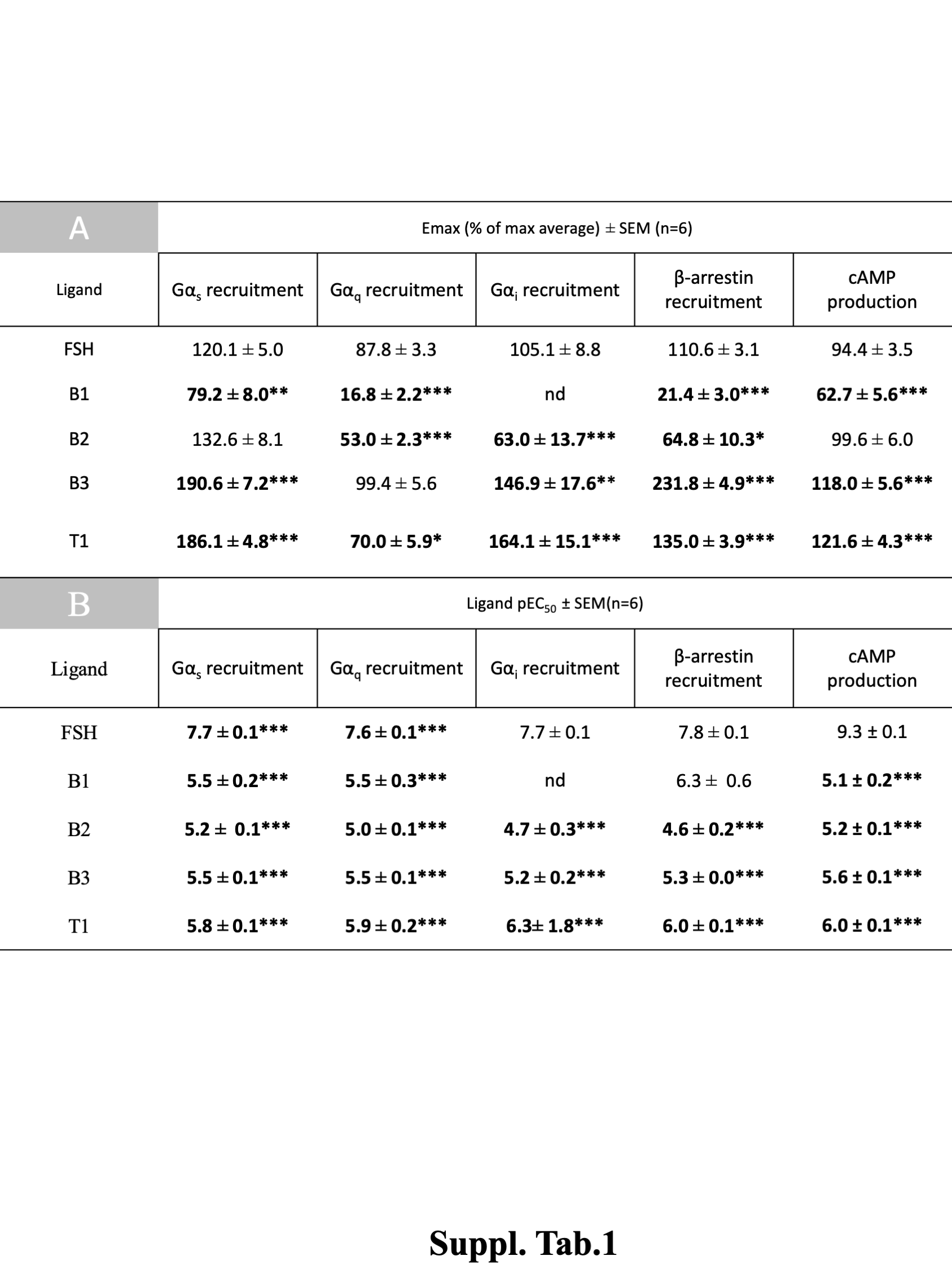

Supplement: Supplementary file 1 [file ijms-22-09850-s001.zip › Supplementary figures_De Pascali et al_/Suppl. Tab. 1.tiff]

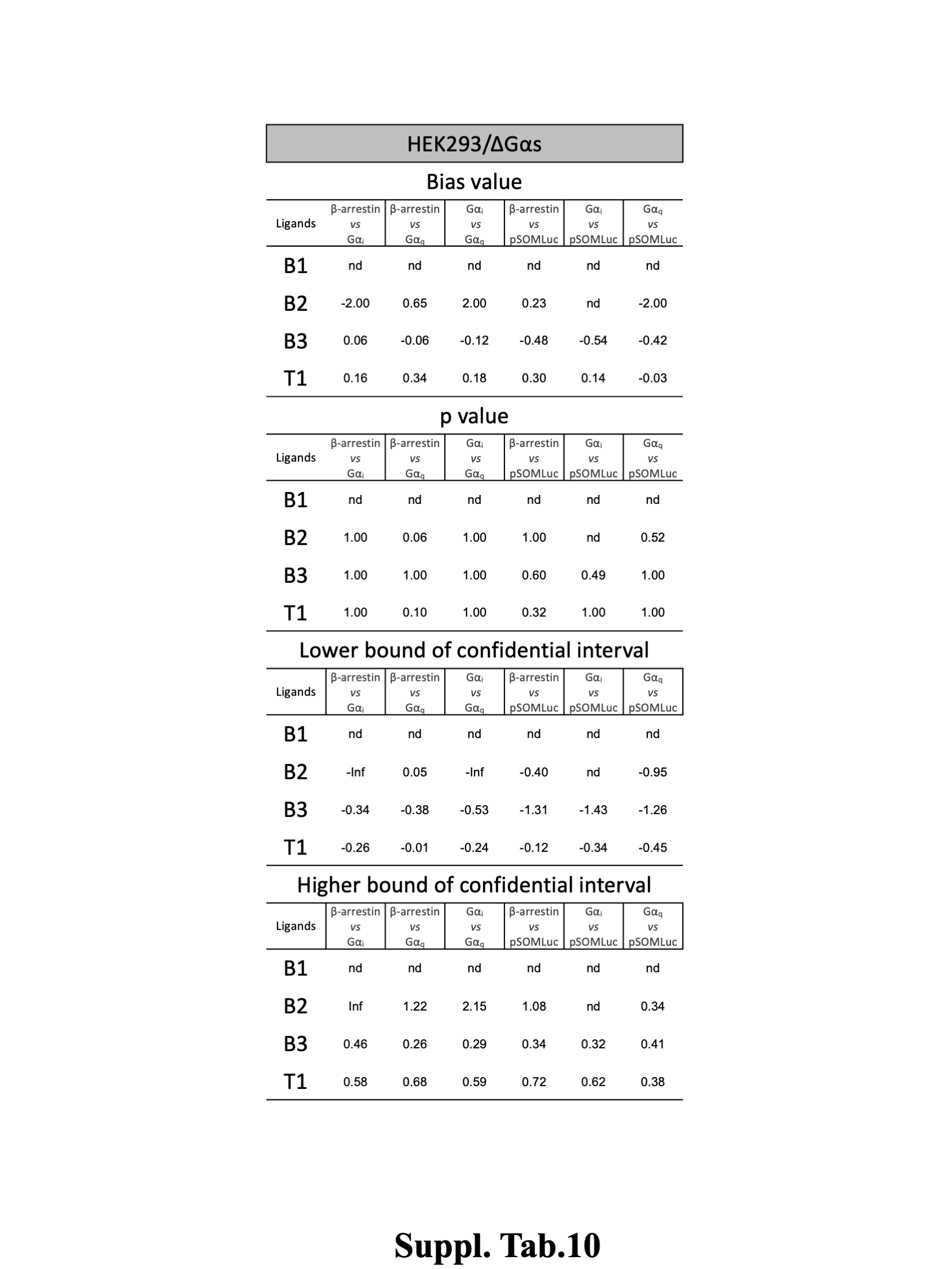

Supplement: Supplementary file 1 [file ijms-22-09850-s001.zip › Supplementary figures_De Pascali et al_/Suppl. Tab. 10.tiff]

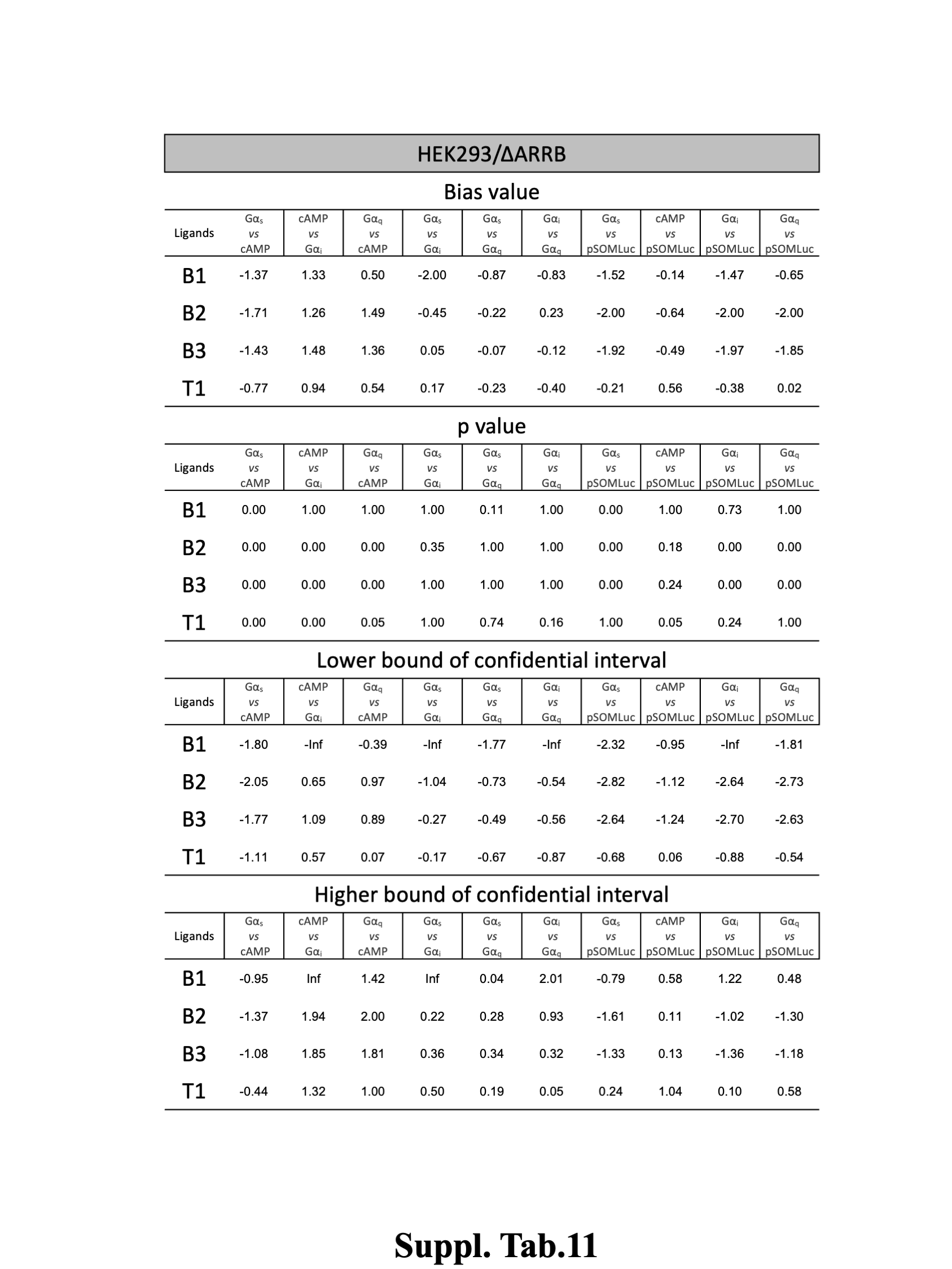

Supplement: Supplementary file 1 [file ijms-22-09850-s001.zip › Supplementary figures_De Pascali et al_/Suppl. Tab. 11.tiff]

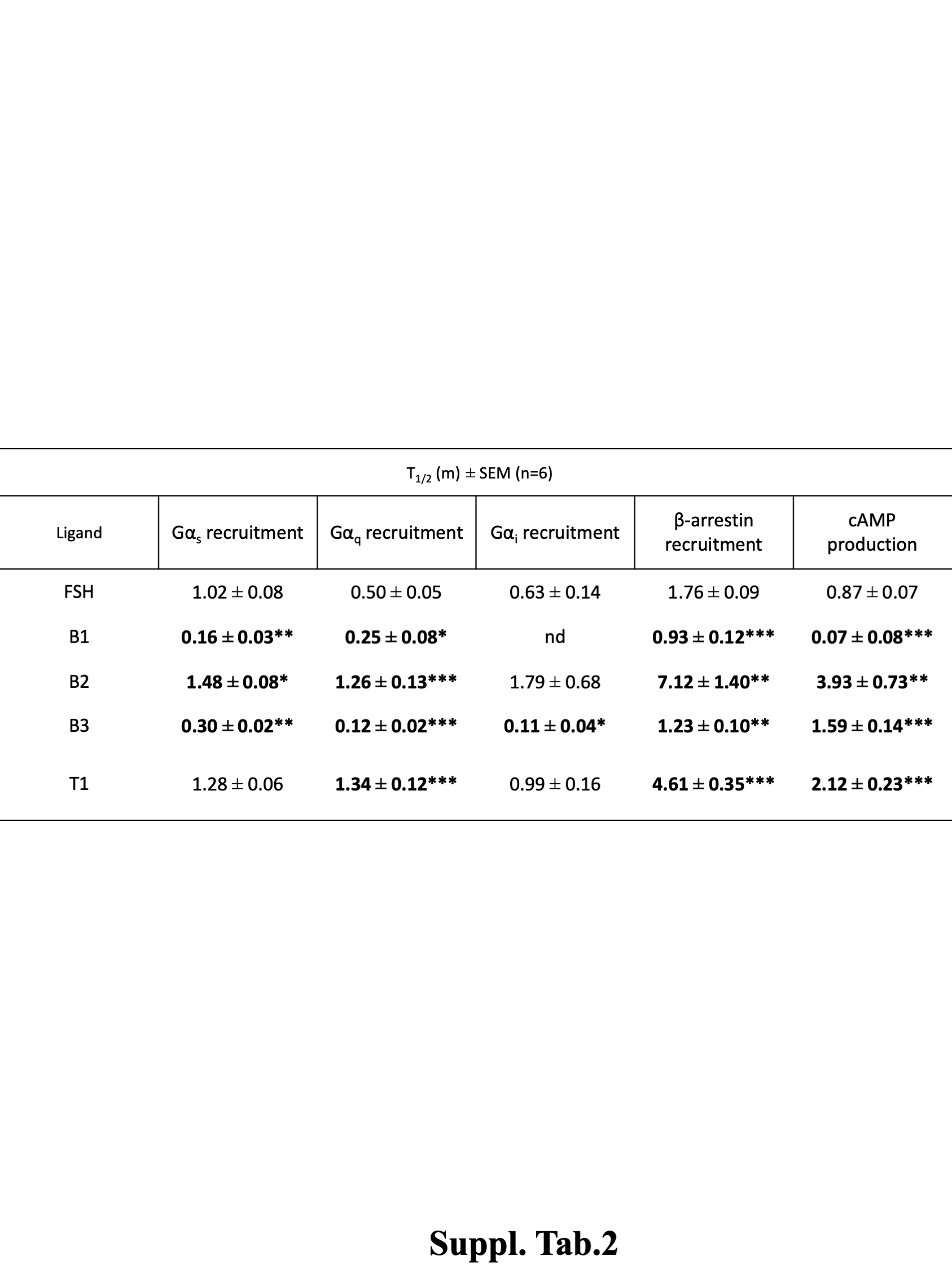

Supplement: Supplementary file 1 [file ijms-22-09850-s001.zip › Supplementary figures_De Pascali et al_/Suppl. Tab. 2.tiff]

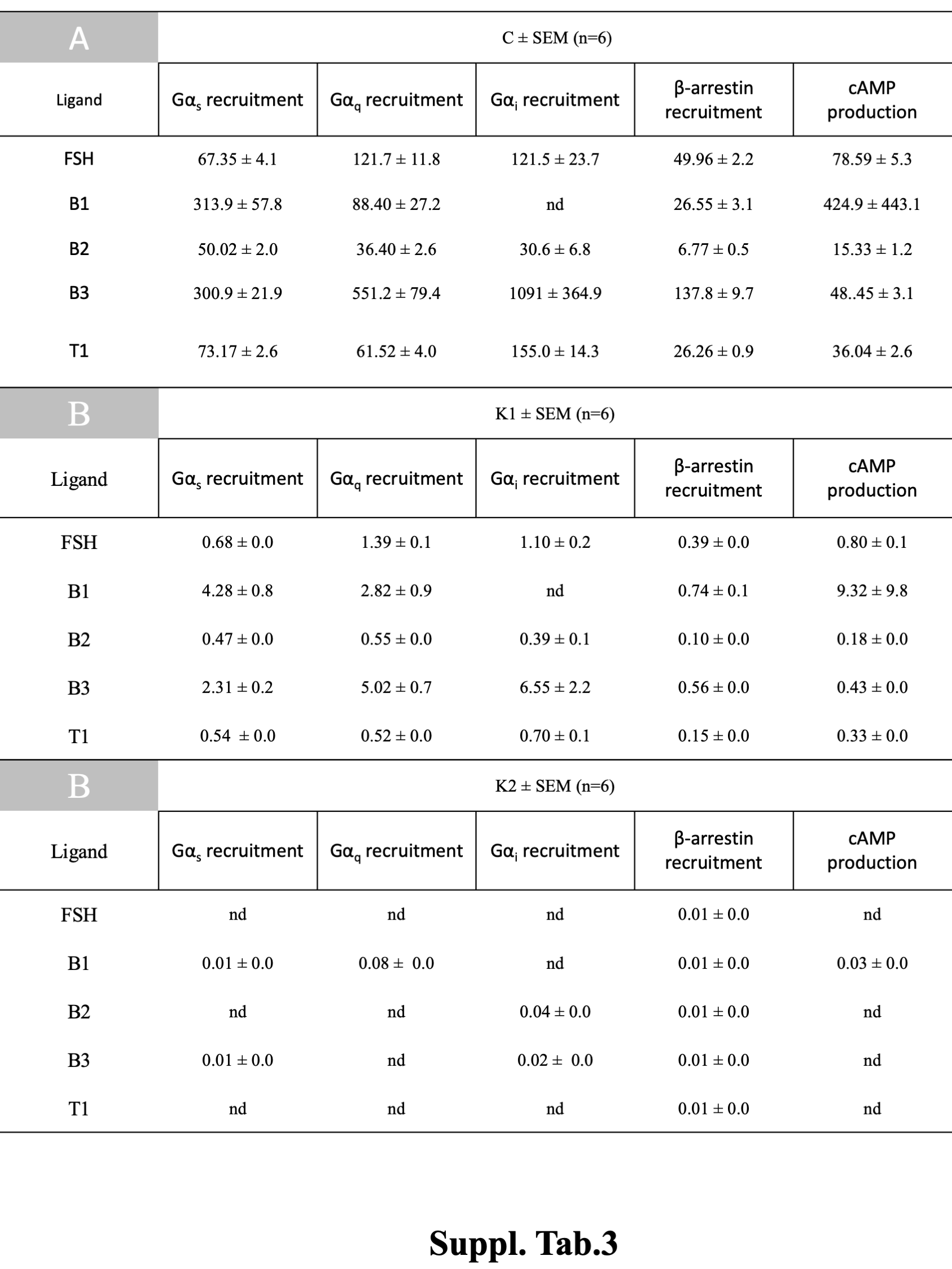

Supplement: Supplementary file 1 [file ijms-22-09850-s001.zip › Supplementary figures_De Pascali et al_/Suppl. Tab. 3.tiff]

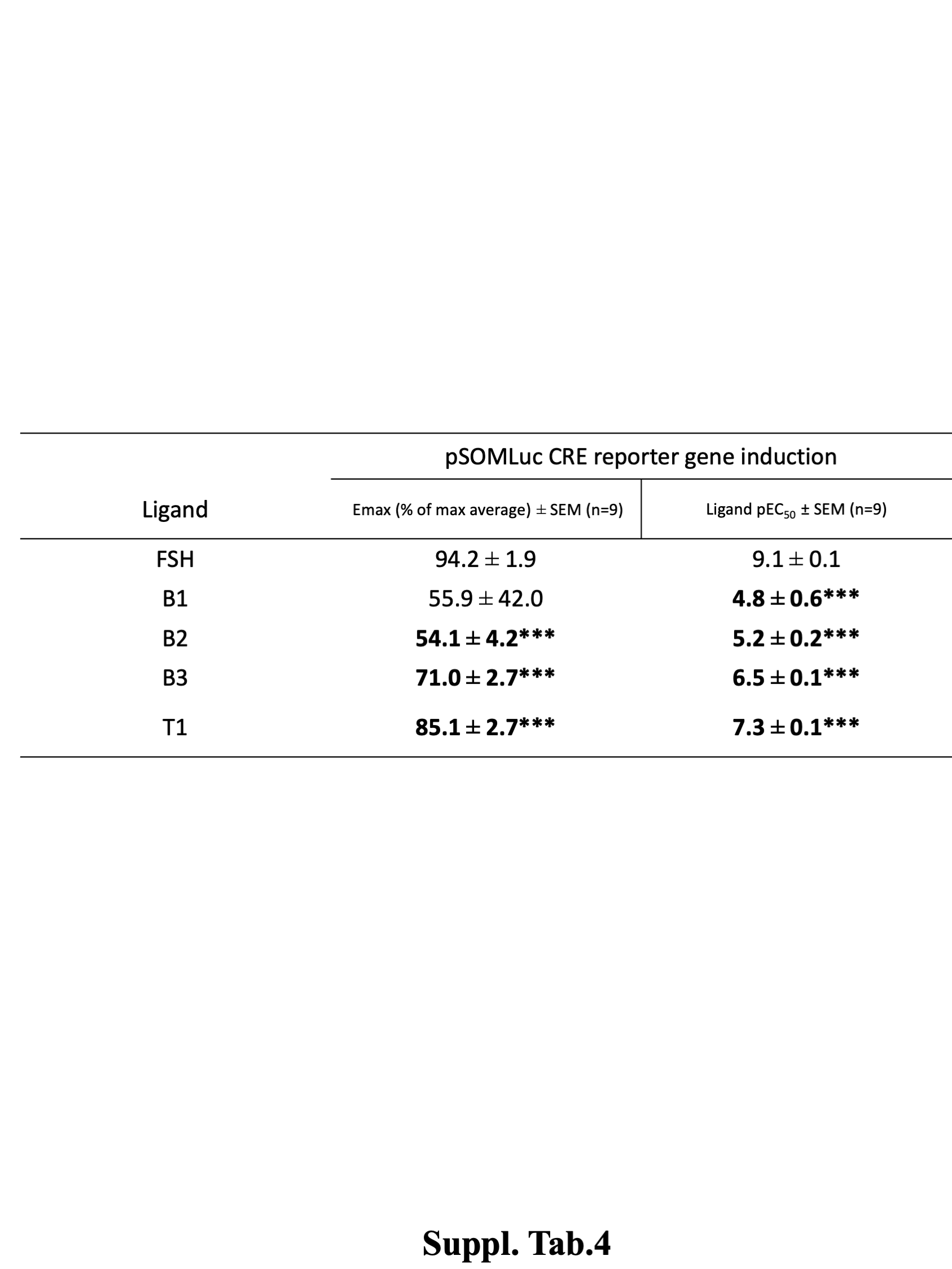

Supplement: Supplementary file 1 [file ijms-22-09850-s001.zip › Supplementary figures_De Pascali et al_/Suppl. Tab. 4.tiff]

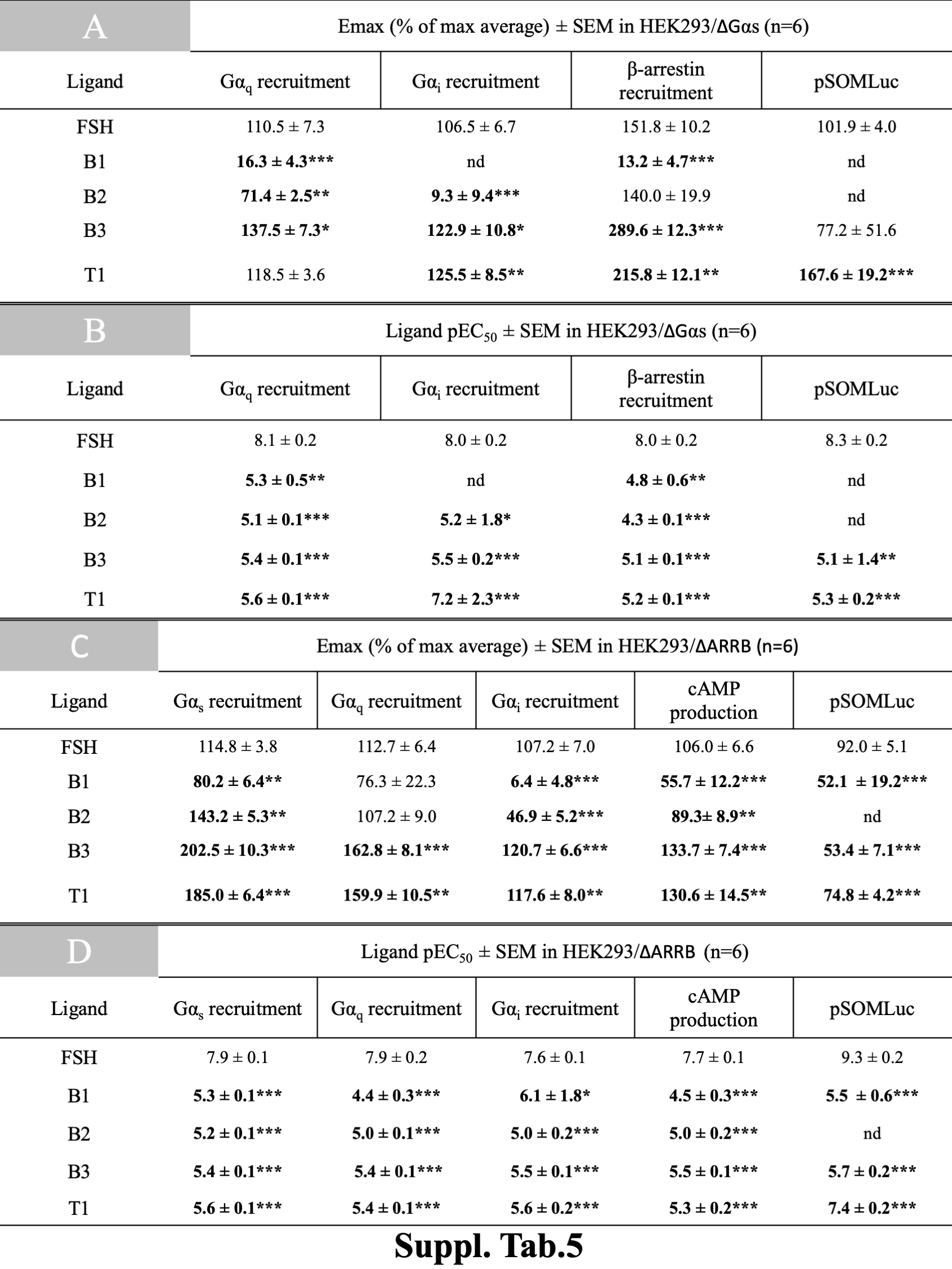

Supplement: Supplementary file 1 [file ijms-22-09850-s001.zip › Supplementary figures_De Pascali et al_/Suppl. Tab. 5.tiff]

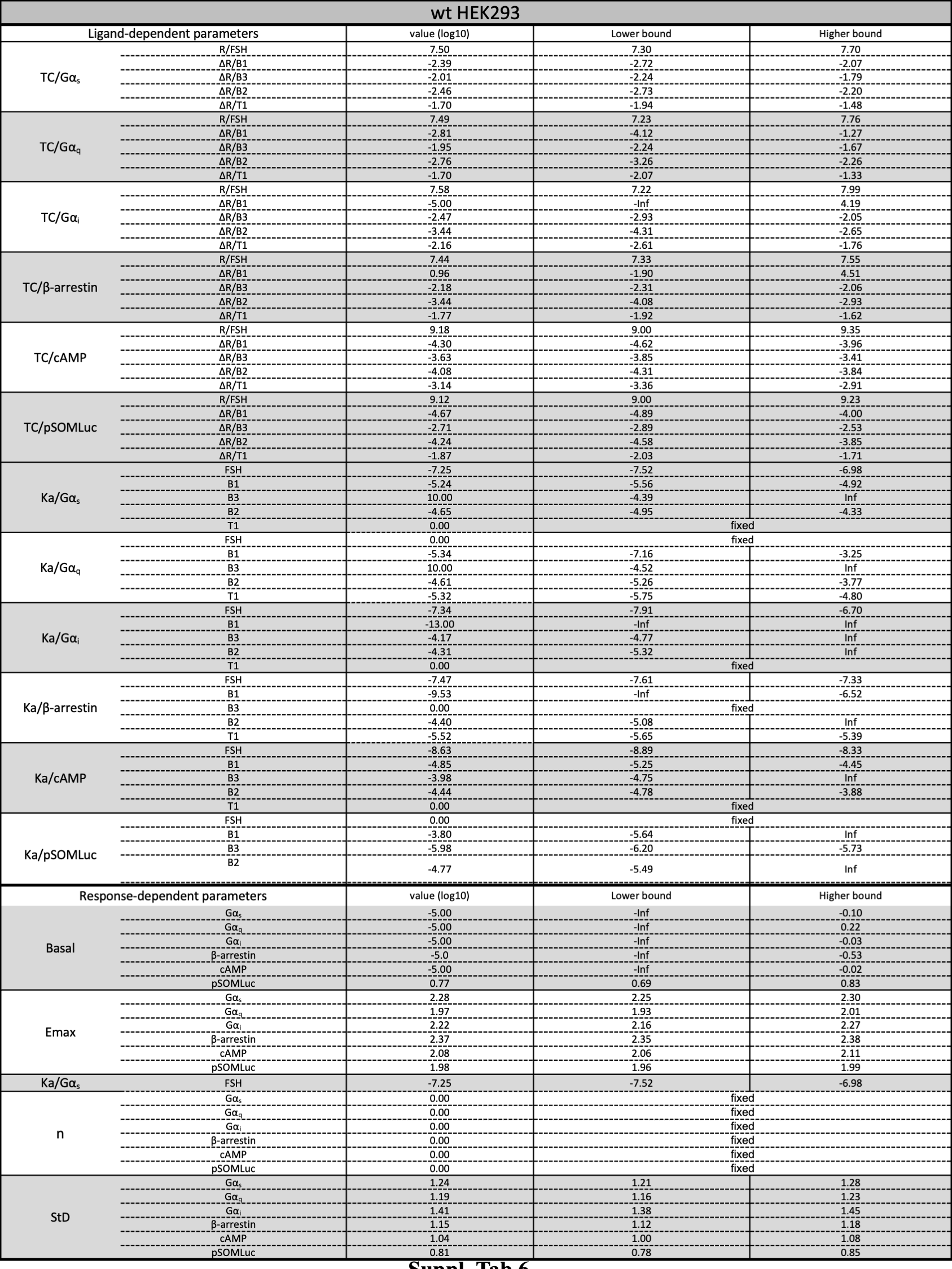

Supplement: Supplementary file 1 [file ijms-22-09850-s001.zip › Supplementary figures_De Pascali et al_/Suppl. Tab. 6.tiff]

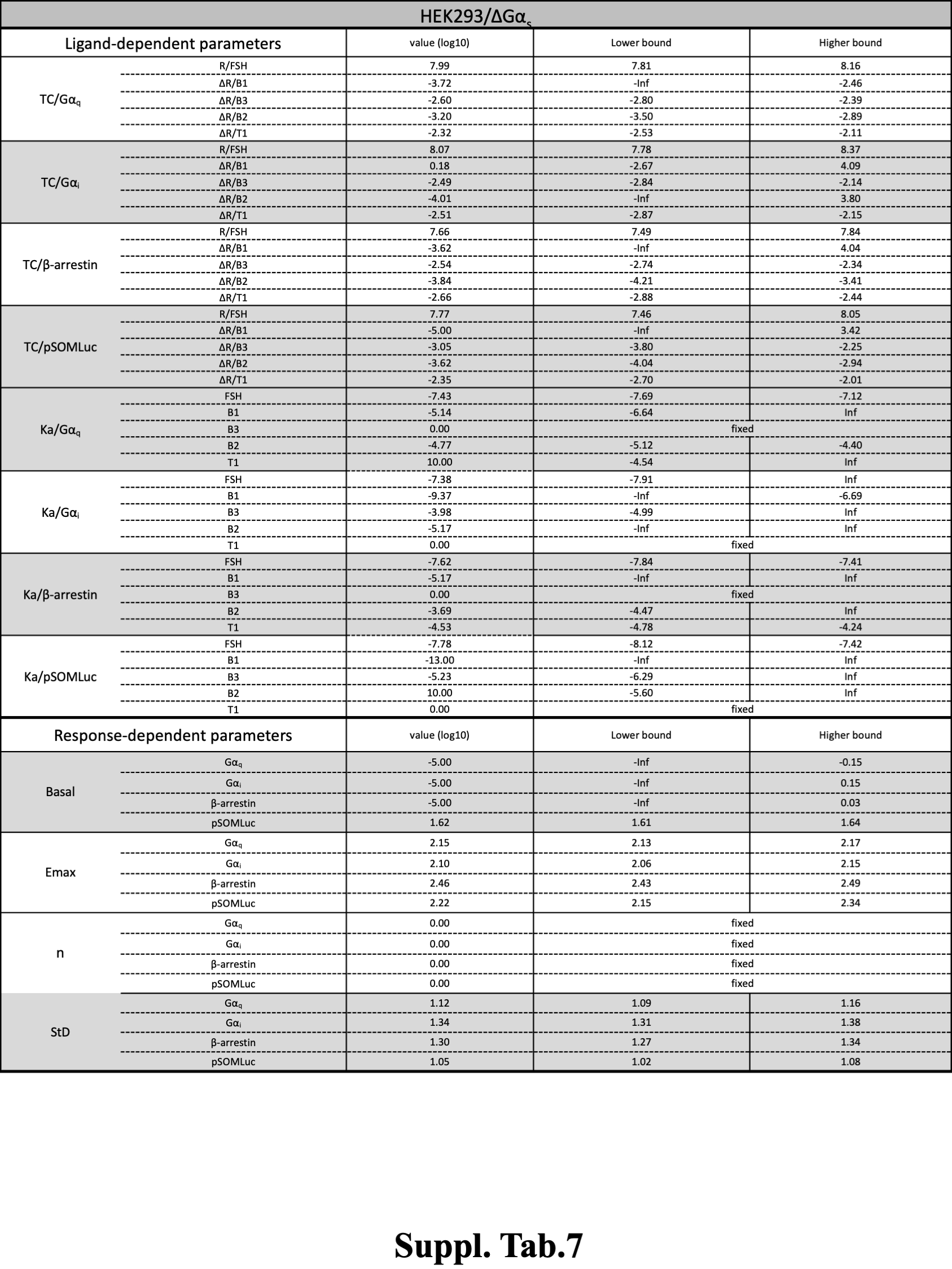

Supplement: Supplementary file 1 [file ijms-22-09850-s001.zip › Supplementary figures_De Pascali et al_/Suppl. Tab. 7.tiff]

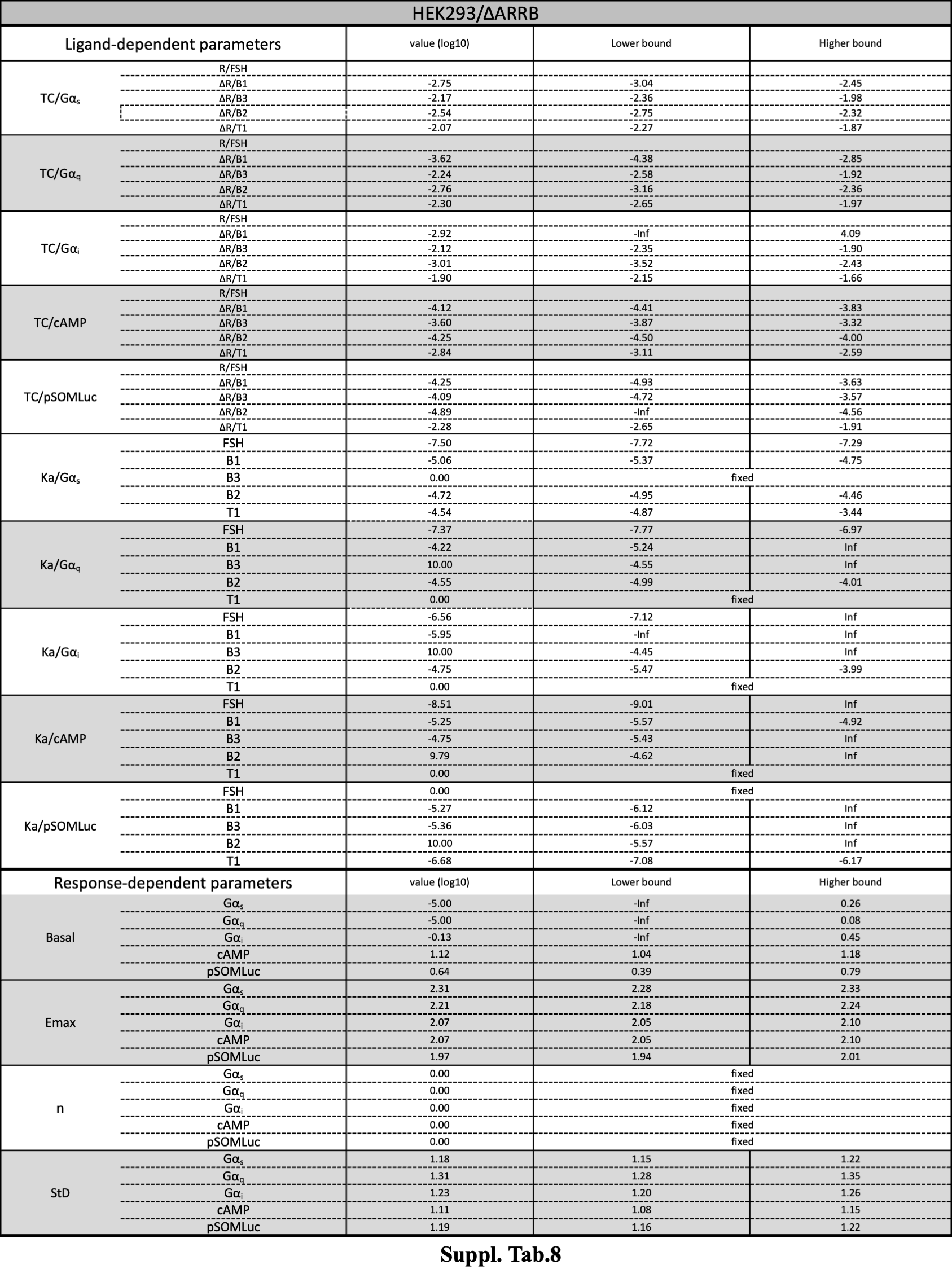

Supplement: Supplementary file 1 [file ijms-22-09850-s001.zip › Supplementary figures_De Pascali et al_/Suppl. Tab. 8.tiff]

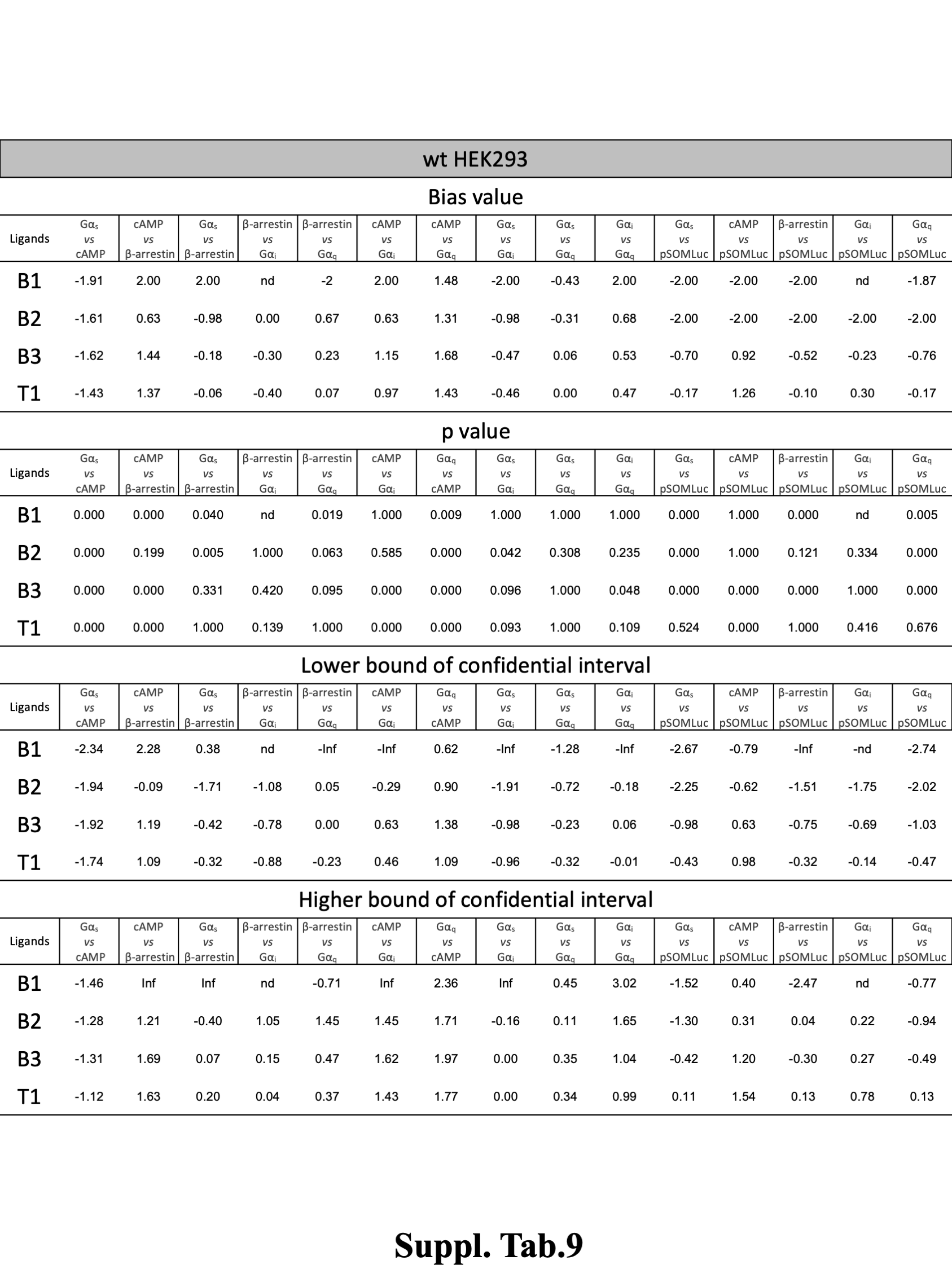

Supplement: Supplementary file 1 [file ijms-22-09850-s001.zip › Supplementary figures_De Pascali et al_/Suppl. Tab. 9.tiff]
